# Nuclear Spin–Spin Coupling Density Functions: Through-Bond and Through-Space Interactions

**DOI:** 10.1021/acs.jpca.5c03609

**Published:** 2026-02-24

**Authors:** Paolo Lazzeretti, Francesco Ferdinando Summa, Guglielmo Monaco, Riccardo Zanasi

**Affiliations:** Dipartimento di Chimica e Biologia “A. Zambelli”, 19028 Università degli Studi di Salerno, via Giovanni Paolo II 132, 84084 Fisciano, SA, Italy

## Abstract

A new method based on the solution of time-independent standard response equation has been developed for the calculation of spin–spin coupling density functions, entirely in the atomic orbital basis at both HF and DFT (GGA and hybrid GGA) level of theory. The study is not limited to the Fermi contact alone, but also includes all four Ramsey terms, which have sometimes been shown to be non-negligible. The *current density* induced by nuclear magnetic dipoles represents the leading motif followed in the development of the theory. A few molecules have been analyzed in detail. The mechanism of spin polarization can be visualized, and the distinction between through-space and through-bond interactions can now be understood in terms of all four Ramsey contributions.

## Graphical abstract

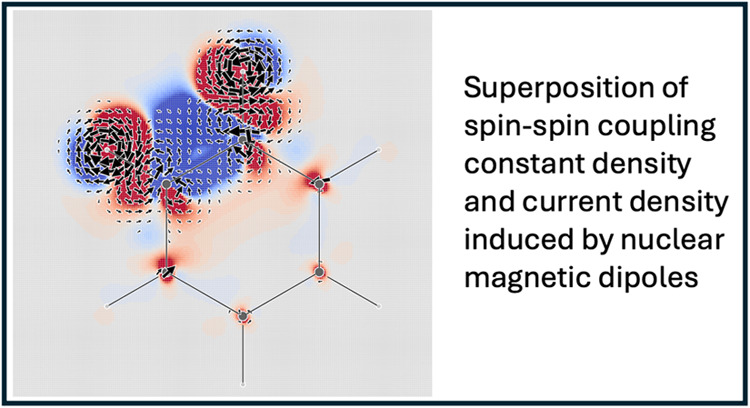

## Introduction

In nuclear magnetic resonance (NMR) spectroscopy, the spin–spin coupling between two nuclear magnetic dipoles,^1–10^ which are separated by more than three consecutive bonds in saturated molecules, is possibly exerted through space (TS) instead of through the bonds (TB), as first suggested in 1961 by Davis, Lutz, and Roberts.^11^ A semiempirical approach, in which such an effect is essentially biased by the Fermi contact interaction of nuclear and electron spins, has been proposed by Buckingham and Cordle,^12^ assuming that TS interaction is proportional to the atomic valence *s* electron density at coupled nuclei.

A review by Hilton and Sutcliffe^13^ on TS scalar spin–spin coupling has appeared in 1975. Contreras and Peralta,^14^ and Hierso^15^ analyzed a series of interesting topics concerning this mechanism. A more recent paper, discussing several issues from rigid intramolecular cases to short-lived van der Waals complexes, has recently been reported by Saielli.^16^ Other results have been reported more recently.^17–20^

The present investigation aims to propose suitable tools for visualizing the actual path, TS or TB, whereby nuclear coupling takes place. Previous attempts in this direction have been made by Malkina and Malkin, who reported maps of “coupling energy density” (CED),^21^ also considered by Gräfenstein and Cremer,^22–24^ Cremer and Gräfenstein.^25^ The calculation and visualization of the spin–spin coupling constant density at relativistic DFT level, adopting the Gordon’s decomposition have been recently reported by Komorovsky et al.^26^

The theory of *indirect* (i.e., electron coupled) spin–spin reciprocal influence between two nuclei of a molecule^1,2,4,7,8,10,27^ has been recast in terms of current densities induced by nuclear magnetic dipoles, considered as “phenomenological” (classical) quantities, in a previous paper.^28^ The key argument justifying such a reformulation is provided by the Hirschfelder concept of *subobservable*, characterizing the basic ideas of charge and current density.^29^

This alternative interpretation has been achieved via a series of simple relationships, describing the polarization of charge distribution and the induction of current density in the electrons of a molecule in the presence of external magnetic fields or intramolecular magnetic dipoles at the nuclei.^30^ The computational procedures are outlined in the Section discussing the theoretical approach, which makes use of assumptions of classical electromagnetism.^31^ In particular, the description of nuclear spin–spin coupling arrived at via current densities is fully consistent with the Biot-Savart law (BSL).^31^

Accordingly, the coupling interaction $K\left(m_{I},m_{J}\right)$ , typically modeled by a nonsymmetric second-rank tensor^3–5^ independent of the magnetic dipoles, arises from superposition (interference) of two current-density vector fields, induced in the electrons of a molecule by the nuclear magnetic dipoles ***m***_*I*_ and ***m***_*J*_.^32^ Another instrument, very useful to determine the actual path whereby coupled nuclear magnetic dipoles “talk to one another”, is provided by maps of coupling density functions,^33–36^ $\kappa^{\mathrm{IJ}}\left(r\right)$ , which clearly show molecular domains where larger interactions occur, together with corresponding plots of intensity (modulus) of electron current density. Thus, coupling-density maps and current-density maps, to which the former are directly connected by a geometry-dependent scaling,^36^ yield fundamental complementary information on the nuclear coupling phenomenon, e.g., transmission pathways^11,12^ and mechanisms.

Of course, the problem of computing quantum-mechanical current densities as functions of position ***r*** requires appropriate procedures,^37^ within a given approximation. In any case, the equations involving these quantities are very general and formally identical with their classical analogues.^31^

A generally accepted criterion for the interpretation of NMR nuclear spin–spin coupling constants, usually dominated by the Fermi contact (FC) mechanism, relies on the Heisenberg–Dirac–Van Vleck (HDVV) vector model.^38–41^ Within the HDVV description, the electron spin polarization induced at the site of a perturbing nucleus by the FC interaction gives rise to a clear-cut electron spin density^42^ in the proximity of the other nuclei of the molecule: if the number of bonds separating the coupled nuclei along a given path is odd (even), spin polarization about the coupled nucleus is reversed (preserved), determining a positive (negative) coupling constant. Of course, no prediction of the coupling magnitude is provided by this simple rule, although computational experience shows that the FC contribution decays quite briskly (in fact exponentially) with the distance between the coupled nuclear dipoles, see for instance theoretical and experimental data for *J*(^13^C^13^C) and *J*(^13^C^1^H) in a few simple molecules.^43^

## Theoretical Approach

For a molecule with *n* electrons and *N* clamped nuclei, charge, mass, position, canonical and angular momentum of the *k*th electron are indicated, in the configuration space, by −*e*, *m*_e_, ***r***_*k*_, ***p* ^**_*k*_, ***l* ^**_*k*_ = ***r***_*k*_ × ***p* ^**_*k*_, *k* = 1, 2, ..., *n*, using boldface letters for electronic operators. Analogous quantities for nucleus *I* are, for instance, *Z*_*I*_ *e*, *M*_*I*_, ***R***_*I*_, etc. for *I* = 1, 2, ..., *N*. We suppose that *N*′ nuclei are endowed with a magnetic dipole ***m***_*I*_ = γ_*I*_ ℏ ***I***_*I*_, defined via magnetogyric ratio γ_*I*_ and spin ℏ ***I***_*I*_ of nucleus *I*. The imaginary unit is represented by a Roman i. Throughout this paper, SI units are used and standard tensor formalism is employed, e.g., the Einstein convention of implicit summation over two repeated Greek indices is in force. The third-rank pseudotensor defined by Ricci and Levi–Civita is indicated by ϵ_αβγ_. Capitals denote *n*-electron vector operators, e.g., the operator representing the electric field exerted by the *k*th electron upon the *I*th nucleus is expressed by

1 $${Ê}_{I}^{k}=\frac{1}{4\pi {\text{ϵ}}_{0}}e\frac{r_{k}-R_{I}}{|r_{k}-R_{I}{|}^{3}},,{Ê}_{I}^{n}=\sum_{k=1}^{n}{Ê}_{I}^{k}$$

where −*e* the charge of an electron, and the corresponding operator for the electric field on the *k*th electron exerted by nucleus *I*, with charge *Z*_*I*_ *e*, is ***E* ^**_*k*_^*I*^ = *Z*_*I*_ ***E* ^**_*I*_^*k*^. The Hermitian operators for the magnetic field on nucleus *I* arising from orbital electron motion are

2 $${\mathrm{B̂}}_{I}^{k}=-\frac{e}{m_{e}}{\mathrm{M̂}}_{I}^{k},,{\mathrm{B̂}}_{I}^{n}=\sum_{k=1}^{n}{\mathrm{B̂}}_{I}^{k}$$

with

3 $${\mathrm{M̂}}_{I}^{k}=\frac{\mu_{0}}{4\pi }\frac{\left(r_{k}-R_{I}\right)}{|r_{k}-R_{I}{|}^{3}}\times {\mathrm{p̂}}_{k}\equiv \frac{\mu_{0}}{4\pi }\frac{{\mathrm{l̂}}_{k}\left(R_{I}\right)}{|r_{k}-R_{I}{|}^{3}}$$

denoting the angular momentum operator of the *k*th electron with respect to the origin ***R***_*I*_ by ***l* ^**_*k*_(***R***_***I***_), and using the relationship μ_0_ϵ_0_ *c* ^2^ = 1, which connects the permeability of vacuum (magnetic constant), the vacuum electric permittivity (electric constant) and the speed of light in vacuum.^44^

Components are specified by Greek letters, e.g., for the *reduced coupling constant* $K^{I_{\alpha }J_{\beta }}$ , measured in T^2^ J^–1^ ≡ N A^–2^ m^–3^ in SI units,^45,46^ is related to *J* ^*I*_α_ *J*_β_^, the same quantity usually expressed in hertz by NMR spectroscopists:^3,4,47^

4 $$K^{I_{\alpha }J_{\beta }}={\frac{\partial^{2}W^{m_{I}m_{J}}}{\partial m_{I_{\alpha }}\partial m_{J_{\beta }}}|}_{m_{I},m_{J}\to 0}=\frac{2\pi J^{I_{\alpha }J_{\beta }}}{\hbar \gamma_{I}\gamma_{J}}$$

Accordingly, the electron-coupled interaction energy of the nuclear magnetic dipoles ***m***_*I*_ and ***m***_*J*_ is

5 $$W^{m_{I}m_{J}}=m_{I_{\alpha }}K^{I_{\alpha }J_{\beta }}m_{J_{\beta }}=-m_{I_{\alpha }}\left\langle {\mathrm{B̂}}_{I_{\alpha }}^{n}\right\rangle$$

In virtue of eq 5, the *n* molecular electrons perturbed by the nuclear magnetic dipole ***m***_*J*_ induce a (time-averaged) magnetic field at nucleus *I*, which can be described via the coupling tensor ⟨*B̂*_*I*_α__^*n*^⟩.^48^ The vector potential at position point ***r***, associated with the magnetic dipole ***m***_*I*_ at ***R***_*I*_, is

6 $$A^{m_{I}}\left(r-R_{I}\right)=\frac{\mu_{0}}{4\pi }\frac{m_{I}\times \left(r-R_{I}\right)}{|r-R_{I}{|}^{3}}$$

The Hamiltonian operator for the *k*th electron of the molecule is cast in the form

7 $${ĥ}_{k}={ĥ}_{k}^{\left(0\right)}+{ĥ}_{k}^{m_{I}}+{ĥ}_{k}^{m_{I}m_{J}}+{ĥ}_{k}^{\mathrm{SD}}+{ĥ}_{k}^{F}$$

where *ĥ*_*k*_^(0)^ = (1/2*m*_e_) ***p* ^**_*k*_^2^ + *V̂*_*k*_, with ***p* ^**_*k*_ = −iℏ**∇**_*k*_ is the canonical momentum, and *V̂*_*k*_ includes one- and two-body Coulomb terms. The spin–orbit interaction (SOC)^49^ is expected to yield small third-order contributions to the Fermi contact contribution to the coupling tensor (eq 4).^4,50^

The perturbing operators,^2,4^ to first and second order, are the nuclear spin/electron orbit interaction, which contains terms linear and bilinear in the magnetic dipoles,

8 $${ĥ}_{k}^{m_{I}}=-m_{I}\cdot {\mathrm{B̂}}_{I}^{k}$$

9 $${ĥ}_{k}^{m_{I}m_{J}}=\frac{1}{m_{e}c^{4}}m_{I_{\alpha }}m_{J_{\beta }}\left({Ê}_{I_{\gamma }}^{k}{Ê}_{J_{\gamma }}^{k}\delta_{\alpha \beta }-{Ê}_{J_{\alpha }}^{k}{Ê}_{I_{\beta }}^{k}\right)$$

mixing into one another in a gauge transformation.^51^

The one-electron spin-dipolar (SD) Hamiltonian (SD stands for spin-dipolar, not to be confused with spin density) can be written as

10 $${ĥ}_{k}^{\mathrm{SD}}=-B_{k\alpha }^{\mathrm{SD}}{\mathrm{m̂}}_{k\alpha }^{\left(s\right)}=\frac{\mu_{0}}{4\pi }\mu_{B}m_{I_{\beta }}\frac{3\left(r_{k\alpha }-R_{I_{\alpha }}\right)\left(r_{k\beta }-R_{I_{\beta }}\right)-\delta_{\alpha \beta }{\left|r_{k}-R_{I}\right|}^{2}}{{\left|r_{k}-R_{I}\right|}^{5}}{\mathrm{\sigma ̂}}_{k\alpha }$$

where the magnetic moment of one electron is

11 $${\mathrm{m̂}}^{\left(s\right)}=-g_{e}\mu_{B}\left(ŝ/\hbar \right)\approx -\frac{e}{m_{e}}ŝ=-\mu_{B}\mathrm{\sigma ̂}$$

and the Fermi contact operator is defined by the Poisson equation,^36^ i.e.,

12 $${ĥ}_{k}^{F}=-B_{k\alpha }^{F}{\mathrm{m̂}}_{k\alpha }^{\left(s\right)}=\frac{2}{3}\mu_{0}\mu_{B}m_{l_{\alpha }}\delta \left(r_{k}-R_{I}\right){\mathrm{\sigma ̂}}_{k\alpha },,{\mathrm{m̂}}_{k\alpha }^{\left(s\right)}=-\frac{e}{m_{e}}{ŝ}_{k\alpha }$$

In eqs 10–12, the Bohr magneton is μ_B_= *e*ℏ/2*m*_e_ = 9.274 010 0657(29) × 10^–24^ J T^–1^,^44^ and, for the electron spin, the most accurate (absolute) value for the *g*_e_ factor has been experimentally determined to be 2.002 319 304 360 92(36).^44^

Within the Ramsey^2^ approach, based on the Rayleigh–Schrödinger perturbation theory (RSPT), the operator for the total magnetic field, acted by the *n* electrons of a molecule upon nucleus *I*, is expressed by the sum of vector operators:

13 $${\mathrm{B̂}}_{I}^{n}={\mathrm{B̂}}_{I}^{\left(1a\right)n}+{\mathrm{B̂}}_{I}^{\left(1b\right)n}+{\mathrm{B̂}}_{I}^{\left(2\right)n}+{\mathrm{B̂}}_{I}^{\left(3\right)n}$$

with ${\mathrm{B̂}}_{I}^{\left(1b\right)n}=\hat{B_{I}^{n}\;}$ given by eq 2. Thus, the Hamiltonian *Ĥ* ^m_Iα_^ *m*_*I*_α__ of the molecule in the presence of intrinsic nuclear magnetic dipoles, can be written in terms of reduced terms

14 $${Ĥ}^{m_{I_{\alpha }}}={Ĥ}_{\alpha }^{\left(1a\right)}+{Ĥ}_{\alpha }^{\left(1b\right)}+{Ĥ}_{\alpha }^{\left(2\right)}+{Ĥ}_{\alpha }^{\left(3\right)}$$

Eventually, the Ramsey Hamiltonian operators, for the whole set of perturbing nuclear magnetic dipoles ***m***_***I***_, *I* = 1, ..., *N*′, are given by

15 $${Ĥ}^{\left(1a\right)}\equiv {Ĥ}_{\mathrm{DSO}}={\left(\frac{\mu_{0}}{4\pi }\right)}^{2}\frac{e^{2}}{2m_{e}}\sum_{I,J\neq I}^{N^{\prime }}m_{I_{\alpha }}{Û}_{I_{\alpha }J_{\beta }}^{n}m_{J_{\beta }}$$

16 $${Ĥ}^{\left(1b\right)}\equiv {Ĥ}_{\mathrm{PSO}}=-\sum_{I=1}^{N^{\prime }}m_{I_{\alpha }}{\mathrm{B̂}}_{I_{\alpha }}^{n}=\frac{e}{m_{e}}\sum_{I=1}^{N^{\prime }}m_{I_{\alpha }}{\mathrm{M̂}}_{I_{\alpha }}^{n}$$

17 $${Ĥ}^{\left(2\right)}\equiv {Ĥ}_{\mathrm{SD}}=\frac{\mu_{0}}{4\pi }\mu_{B}\sum_{I}^{N^{\prime }}m_{I_{\alpha }}{\mathrm{T̂}}_{I_{\alpha }}^{n}$$

18 $${Ĥ}^{\left(3\right)}\equiv {Ĥ}_{\mathrm{FC}}=\frac{2}{3}\mu_{0}\mu_{B}\sum_{I=1}^{N^{\prime }}m_{I_{\alpha }}{\mathrm{F̂}}_{I_{\alpha }}^{n}$$

introducing the auxiliary operators

19 $${Û}_{I_{\alpha }J_{\beta }}^{n}=\sum_{k=1}^{n}r_{\mathrm{kI}}^{-3}r_{\mathrm{kJ}}^{-3}\left(r_{kI_{\gamma }}r_{kJ_{\gamma }}\delta_{\alpha \beta }-r_{kJ_{\alpha }}r_{kI_{\beta }}\right)$$

20 $${\mathrm{T̂}}_{I_{\alpha }}^{n}=\sum_{k=1}^{n}\left[\left(3r_{kI_{\alpha }}r_{kI_{\gamma }}-r_{\mathrm{kI}}^{2}\delta_{\alpha \gamma }\right)r_{\mathrm{kI}}^{-5}\right]{\mathrm{\sigma ̂}}_{k\gamma }$$

21 $${\mathrm{F̂}}_{I_{\alpha }}^{n}=\sum_{k=1}^{n}\delta \left(r_{\mathrm{kI}}\right){\mathrm{\sigma ̂}}_{k\alpha }$$

where ***r***_*kI*_ = ***r***_*k*_ – ***R***_*I*_ and **σ̂**_*k*_ is a Pauli matrix for the *k*th electron. Allowing for the definition of the polarization propagator^52^

22 $${\left\{Â,\mathrm{B̂}\right\}}_{-1}=\frac{2}{\hbar }\sum_{j\neq a}\omega_{\mathrm{ja}}^{-1}R\left\{\langle a\left|Â\left|j\right\rangle \langle j\right|\mathrm{B̂}\left|a\right\rangle \right\}\equiv -\langle {\left\langle A;B\right\rangle \rangle }_{\omega =0}$$

for eqs 15, 16, and 19–21, the contributions to the reduced nuclear spin–spin coupling tensors are expressed in the form

23 $$K^{\left(1a\right)I_{\alpha }J_{\beta }}={\left(\frac{\mu_{0}}{4\pi }\right)}^{2}\frac{e^{2}}{m_{e}}\left\langle a\right|{Û}_{I_{\alpha }J_{\beta }}^{n}\left|a\right\rangle \equiv K_{\mathrm{DSO}}^{I_{\alpha }J_{\beta }}$$

24 $$K^{\left(1b\right)I_{I_{\alpha }J_{\beta }}}=-{\left(\frac{e}{m_{e}}\right)}^{2}{\left\{{\mathrm{M̂}}_{I_{\alpha }}^{n},{\mathrm{M̂}}_{J_{\beta }}^{n}\right\}}_{-1}=-{\left\{{\mathrm{B̂}}_{I_{\alpha }}^{n},{\mathrm{B̂}}_{J_{\beta }}^{n}\right\}}_{-1}\equiv K_{\mathrm{PSO}}^{I_{\alpha }J_{\beta }}$$

25 $$K^{\left(2\right)I_{\alpha }J_{\beta }}=-{\left(\frac{\mu_{0}}{4\pi }\right)}^{2}\mu_{B}^{2}{\left\{{\mathrm{T̂}}_{I_{\alpha }}^{n},{\mathrm{T̂}}_{J_{\beta }}^{n}\right\}}_{-1}\equiv K_{\mathrm{SD}}^{I_{\alpha }J_{\beta }}$$

26 $$K^{\left(3\right)I_{\alpha }J_{\beta }}=-{\left(\frac{2\mu_{0}\mu_{B}}{3}\right)}^{2}{\left\{{\mathrm{F̂}}_{I_{\alpha }}^{n},{\mathrm{F̂}}_{J_{\beta }}^{n}\right\}}_{-1}\equiv K_{\mathrm{FC}}^{I_{\alpha }J_{\beta }}$$

27 $$K^{\left(4\right)I_{\alpha }J_{\beta }}=-\frac{\mu_{0}^{2}\mu_{B}^{2}}{6\pi }\left({\left\{{\mathrm{T̂}}_{I_{\alpha }}^{n},{\mathrm{F̂}}_{J_{\beta }}^{n}\right\}}_{-1}+{\left\{{\mathrm{F̂}}_{I_{\alpha }}^{n},{\mathrm{T̂}}_{J_{\beta }}^{n}\right\}}_{-1}\right)\equiv K_{\mathrm{FC}/\mathrm{SD}}^{I_{\alpha }J_{\beta }}$$

The tensors in eqs 23–25 are nonsymmetrical, the tensor in eq 26 is diagonal and isotropic, and the tensor in eq 27, mixing FC and SD contributions, is traceless and symmetric in the indices α and β. Thus, only $K_{\mathrm{FC}}^{I_{x}J_{x}}=K_{\mathrm{FC}}^{I_{y}J_{y}}=K_{\mathrm{FC}}^{I_{z}J_{z}}$ do not vanish.

### Electron Charge Density, Spin Density and Current Densities Induced by Nuclear Magnetic Dipoles

Let us introduce the general definition of the *n*-electron density matrix within the McWeeny–Sutcliffe normalization:^42^

28 $$\gamma \left(x_{1};x_{1}^{\prime }\right)=n\int \Psi \left(x_{1},X_{1}\right)\Psi^{*}\left(x_{1}^{\prime },X_{1}\right)\;dX_{1}$$

denoting by Ψ­(***X***) a wave function which depends on space–spin electronic coordinates ***x***_*k*_ = ***r***_*k*_ ⊗ η_*k*_, *k* = 1, 2, ..., *n*, where

29 $$X_{1}\equiv \left\{x_{2},...,x_{n}\right\},,X=\left\{x_{1},X_{1}\right\},,dX_{1}\equiv \left\{dx_{2},...,dx_{n}\right\}$$

Thus, integrating over dη_1_, one gets from eq 28

30 $$\gamma^{\left(0\right)}\left(r\right)\equiv \gamma^{\left(0\right)}\left(r;r\right)=n\int \Psi_{a}^{\left(0\right)}\left(r,X_{1}\right)\Psi_{a}^{\left(0\right)*}\left(r,X_{1}\right)\;dX_{1}$$

for the reference (ground) state Ψ_*a*_^(0)^ of the molecule. In the absence of external perturbing fields, the electron charge density is ρ^(0)^(***r***) = −*e*γ^(0)^(***r***), and the spin density matrix is obtained by the integral^42^

31 $$Q_{\alpha }\left(r;r^{\prime }\right)=\int_{\eta_{1}^{\prime }=\eta_{1}}{ŝ}_{\alpha }\left(1\right)\gamma \left(x_{1};x_{1}^{\prime }\right)\;d\eta_{1}$$

so that, putting ***r*** = ***r*** ′, we obtain the spin density, described by the axial vector

32 $$Q_{\alpha }\left(r\right)\equiv Q_{\alpha }\left(r;r^{\prime }\right)$$

The probability current density at the position point ***r*** is evaluated by^28^

33 $$j\left(r\right)=\frac{1}{m_{e}}R{\left[\mathrm{\pi ̂}\gamma \left(r;r^{\prime }\right)\right]}_{r\prime =r}+\frac{1}{m_{e}}\nabla \times Q\left(r\right)$$

where **π̂** is the mechanical momentum

34 $$\mathrm{\pi ̂}=\mathrm{p̂}+e\sum_{I=1}^{N^{\prime }}A^{m_{I}}$$

Thus, the electron current density becomes^35,36^ ***J*** (***r***) = −*e* ***j*** (***r***), which can be related to the magnetization density^31^ $M^{m_{I}}\left(r\right)$

35 $$J^{m_{I}}\left(r\right)=\nabla \times M^{m_{I}}\left(r\right),,M^{m_{I}}\left(r\right)=-\frac{e}{m_{e}}Q^{m_{I}}\left(r\right)$$

where ***Q*** ^***m***_*I*_^(***r***) indicates the electron spin density.

Actually, in the presence of intramolecular magnetic dipoles, different types of current density are induced in the electron cloud. Within the Ramsey nomenclature, two of them are cast in the form of diamagnetic and paramagnetic contributions

36 $$J^{\left(1a\right)}\left(r\right)\equiv J_{d}^{m_{I}}\left(r\right)=-\frac{e^{2}}{m_{e}}\gamma^{\left(0\right)}\left(r\right)A^{m_{I}}\left(r-R_{I}\right)$$

37 $$J^{\left(1b\right)}\left(r\right)\equiv J_{p}^{m_{I}}=\frac{\mathrm{ne}}{m_{e}}\int d\eta_{1}\;dX_{1}\;\left\{m_{I}\cdot \Psi_{a}^{\left(1b\right)*}\left(x_{1},X_{1}\right)\mathrm{p̂}\Psi_{a}^{\left(0\right)}\left(x_{1},X_{1}\right)+\Psi_{a}^{\left(0\right)*}\left(x_{1},X_{1}\right)\mathrm{p̂}\Psi_{a}^{\left(1b\right)}\left(x_{1},X_{1}\right)\cdot m_{I}\right\}$$

These terms exchange into one another in a gauge transformation of the vector potential ***A*** ^***m***_*I*_^(***r***), but their sum ***J*** ^***m***_***I***_^ = ***J***_d_^***m***_***I***_^+ ***J***_p_^***m***_***I***_^ remains invariant.^51^ Equation 36 can be expressed via a sum over states formula,^51^ as first shown by Sauer.^53,54^ The spin-dipolar and Fermi contact current densities are defined by

38 $$J^{\left(2\right)}\left(r\right)=-\frac{\mathrm{ne}}{m_{e}}\int d\eta_{1}\;dX_{1}\;\nabla \times \left\{m_{I}\cdot \Psi_{a}^{\left(2\right)*}\left(x_{1},X_{1}\right)ŝ\Psi_{a}^{\left(0\right)}\left(x_{1},X_{1}\right)+\Psi_{a}^{\left(0\right)*}\left(x_{1},X_{1}\right)ŝ\Psi_{a}^{\left(2\right)}\left(x_{1},X_{1}\right)\cdot m_{I}\right\}$$

39 $$J^{\left(3\right)}\left(r\right)=-\frac{\mathrm{ne}}{m_{e}}\int d\eta_{1}\;dX_{1}\;\nabla \times \left\{m_{I}\cdot \Psi_{a}^{\left(3\right)*}\left(x_{1},X_{1}\right)ŝ\Psi_{a}^{\left(0\right)}\left(x_{1},X_{1}\right)+\Psi_{a}^{\left(0\right)*}\left(x_{1},X_{1}\right)ŝ\Psi_{a}^{\left(3\right)}\left(x_{1},X_{1}\right)\cdot m_{I}\right\}$$

The aim of the present paper is to analyze the path of nuclear spins propagation in space. The current density vector induced by the nuclear magnetic dipole ***m***_*I*_ can be defined as

40 $$J^{m_{I}}\left(r\right)=J^{\left(1a\right)}\left(r\right)+J^{\left(1b\right)}\left(r\right)+J^{\left(2\right)}\left(r\right)+J^{\left(3\right)}\left(r\right)$$

that in the complete basis set limit is divergenceless, being^51^

41 $$\int \left(J_{p\alpha }^{m_{I}}+J_{d\alpha }^{m_{I}}\right)\;d^{3}r=\frac{e^{2}}{m_{e}^{2}}\left({\left\{{\mathrm{P̂}}_{\alpha },{\mathrm{M̂}}_{I_{\beta }}^{n}\right\}}_{-1}-\frac{m_{e}}{ec^{2}}{\text{ϵ}}_{\alpha \beta \gamma }\left\langle a\right|{Ê}_{I_{\gamma }}^{n}\left|a\right\rangle \right)m_{I_{\beta }}=0$$

and the divergence of a curl always zero. An analogous expression for the current density vector has been defined in a relativistic framework.^26,55^

The perturbed RSPT wave functions are, for *I* = 1, ..., *N*′,

42 $${\left|\Psi_{a}^{\left(1b\right)}\right\rangle }_{I}=\frac{1}{\hbar }\sum_{j\neq a}\omega_{\mathrm{ja}}^{-1}\left|j\right\rangle \left\langle j\left|{\mathrm{B̂}}_{I}^{n}\right|a\right\rangle$$

43 $${\left|\Psi_{a}^{\left(2\right)}\right\rangle }_{I}=\frac{1}{\hbar }\sum_{j\neq a}\omega_{\mathrm{ja}}^{-1}\left|j\right\rangle \left\langle j\left|{\mathrm{B̂}}_{I}^{\left(2\right)n}\right|a\right\rangle$$

44 $${\left|\Psi_{a}^{\left(3\right)}\right\rangle }_{I}=\frac{1}{\hbar }\sum_{j\neq a}\omega_{\mathrm{ja}}^{-1}\left|j\right\rangle \left\langle j\left|{\mathrm{B̂}}_{I}^{\left(3\right)n}\right|a\right\rangle$$

One can define corresponding current density tensors (CDTs) by differentiating, e.g.,

$$J_{p_{\alpha }}^{m_{I_{\beta }}}\left(r\right)=\frac{\partial J_{p_{\alpha }}^{m_{I}}}{\partial m_{I_{\beta }}}=-\frac{\mathrm{ne}}{m_{e}\hbar }\underset{j\neq a}{\sum }\omega_{\mathrm{ja}}^{-1}\times R\left\{\left\langle a\left|{\mathrm{B̂}}_{I_{\beta }}^{n}\right|j\right\rangle \int \Psi_{j}^{\left(0\right)*}\left(r,X_{1}\right){\mathrm{p̂}}_{\alpha }\Psi_{a}^{\left(0\right)}\left(r,X_{1}\right)\;dX_{1}+\int \Psi_{a}^{\left(0\right)*}\left(r,X_{1}\right){\mathrm{p̂}}_{\alpha }\Psi_{j}^{\left(0\right)}\left(r,X_{1}\right)\;dX_{1}\left\langle j\left|{\mathrm{B̂}}_{I_{\beta }}^{n}\right|a\right\rangle \right\}$$ 45

46 $$J_{d_{\alpha }}^{m_{I_{\beta }}}\left(r\right)=\frac{\partial J_{d_{\alpha }}^{m_{I}}}{\partial m_{I_{\beta }}}=-\frac{\mu_{0}}{4\pi }\frac{e^{2}}{m_{e}}\gamma^{\left(0\right)}\left(r\right){\text{ϵ}}_{\alpha \beta \gamma }\frac{r_{\gamma }-R_{I_{\gamma }}}{|r-R_{I}{|}^{3}}$$

$$J_{p_{\alpha }}^{B_{\beta }}\left(r\right)=\frac{\partial J_{p_{\alpha }}^{B}}{\partial B_{\beta }}=-\frac{\mathrm{ne}}{m_{e}\hbar }\underset{j\neq a}{\sum }\omega_{\mathrm{ja}}^{-1}\times R\left\{\left\langle a\left|{\mathrm{m̂}}_{\beta }\right|j\right\rangle \int \Psi_{j}^{\left(0\right)*}\left(r,X_{1}\right){\mathrm{p̂}}_{\alpha }\Psi_{a}^{\left(0\right)}\left(r,X_{1}\right)\;dX_{1}+\int \Psi_{a}^{\left(0\right)*}\left(r,X_{1}\right){\mathrm{p̂}}_{\alpha }\Psi_{j}^{\left(0\right)}\left(r,X_{1}\right)\;dX_{1}\left\langle j\right|{\mathrm{m̂}}_{\beta }\left|a\right\rangle \right\}$$ 47

48 $$J_{d_{\alpha }}^{B_{\beta }}\left(r\right)=\frac{\partial J_{d_{\alpha }}^{B}}{\partial B_{\beta }}=-\frac{e^{2}}{2m_{e}}{\text{ϵ}}_{\alpha \beta \gamma }r_{\gamma }\gamma^{\left(0\right)}\left(r\right)$$

eqs 47 and 48 define similar CDTs for paramagnetic and diamagnetic contributions to the electron current density induced by a time-independent and spatially uniform magnetic field. They show the analogies between magnetic field and magnetic dipoles, with *m̂*_β_ = (−*e*/2*m*_e_)*L̂*_β_ and *L̂*_β_ = ∑_*k*=1_^*n*^ *l̂*_*k*_β__.

It is useful to recall that the disjoint diamagnetic and paramagnetic components of nuclear spin/electron orbit contributions to coupling constants are not uniquely defined and interchange into each other in a gauge transformation of the vector potential (eq 6).^51^

A CDT analogous to *J*_α_^***m***_*I*_^/∂*m*_*I*_β__ of eqs 45 and 46 can be defined for the current density vector (eq 39).

#### Interchange Theorems

An interchange theorem^36^ states that the electron-coupled interaction energy between a nuclear magnetic shielding and an external magnetic field can be expressed by a spatial integral involving the current density induced by the applied field (the magnetic dipole at nucleus *I*) times the vector potential associated with nucleus *I* (the vector potential of the applied magnetic field), i.e.

49 $$W^{m_{I}B}=-\int A^{m_{I}}\cdot J^{B}\;d^{3}r=-\int A^{B}\cdot J^{m_{I}}\;d^{3}r$$

so that the nuclear magnetic shielding at nucleus *I* is

50 $$\sigma_{\alpha \beta }^{I}=\frac{\partial^{2}W^{m_{I}B}}{\partial m_{I_{\alpha }}\partial B_{\beta }}=\sigma_{\alpha \beta }^{pI}+\sigma_{\alpha \beta }^{dI}$$

where^36^

51 $$\sigma_{\alpha \beta }^{pI}=-{\left\{{\mathrm{B̂}}_{I_{\alpha }}^{n},{\mathrm{m̂}}_{\beta }\right\}}_{-1}=-{\left\{{\mathrm{m̂}}_{\beta },{\mathrm{B̂}}_{I_{\alpha }}^{n}\right\}}_{-1}$$

52 $$\sigma_{\alpha \beta }^{dI}=\frac{e}{2m_{e}c^{2}}\left\langle a\right|\sum_{k=1}^{n}\left(r_{k\gamma }{Ê}_{I_{\gamma }}^{k}\delta_{\alpha \beta }-r_{k\alpha }{Ê}_{I_{\beta }}^{k}\right)\left|a\right\rangle$$

An interchange theorem analogous to eq 49 holds for the indirect coupling energy (see for instance eqs (7.27) and (7.30) of ref 56):

53 $$W^{m_{I}m_{J}}=-\int A^{m_{I}}\cdot J^{m_{J}}\;d^{3}r=-\int A^{m_{J}}\cdot J^{m_{I}}\;d^{3}r$$

For the diamagnetic DSO contribution, since ***J*** ^***m***_*I*_^ ∝ ***A*** ^***m***_*I*_^ and ***J*** ^***m***_*J*_^ ∝ ***A*** ^***m***_*J*_^, this theorem is a mere restatement of the identity ***A*** ^***m***_*I*_^· ***A*** ^***m***_*J*_^ = ***A*** ^***m***_*J*_^· ***A*** ^***m***_*I*_^. The paramagnetic PSO term is cast in the form of the polarization propagator^52^

54 $${\left\{{\mathrm{B̂}}_{I_{\alpha }}^{n},{\mathrm{B̂}}_{J_{\beta }}^{n}\right\}}_{-1}=\frac{2}{\hbar }\sum_{j\neq a}\omega_{\mathrm{ja}}^{-1}R\left\{\left\langle a\left|{\mathrm{B̂}}_{I_{\alpha }}^{n}\right|j\right\rangle \left\langle j\left|{\mathrm{B̂}}_{J_{\beta }}^{n}\right|a\right\rangle \right\}=\frac{1}{\hbar }\sum_{j\neq a}\omega_{\mathrm{ja}}^{-1}\left\{\left\langle a\left|{\mathrm{B̂}}_{I_{\alpha }}^{n}\right|j\right\rangle \left\langle j\left|{\mathrm{B̂}}_{J_{\beta }}^{n}\right|a\right\rangle +\left\langle a\left|{\mathrm{B̂}}_{J_{\beta }}^{n}\right|j\right\rangle \left\langle j\left|{\mathrm{B̂}}_{I_{\alpha }}^{n}\right|a\right\rangle \right\}={\left\{{\mathrm{B̂}}_{J_{\beta }}^{n},{\mathrm{B̂}}_{I_{\alpha }}^{n}\right\}}_{-1}\equiv -\langle {\left\langle {\mathrm{B̂}}_{I_{\alpha }}^{n};{\mathrm{B̂}}_{J_{\beta }}^{n}\right\rangle \rangle }_{\omega =0}$$

which is symmetric in the change of indices *I*_α_ and *J*_β_. Thus, the interchange theorem is proven also for the PSO term via eqs 6 and 45.

#### Coupling Density Functions

Let d ***l*** be an element of length, with position ***r***, in the direction of current flow, and ***R***_*J*_ – ***r*** the vector from d ***l*** to the observation point ***R***_*J*_, where the nucleus *J* is placed. Then the element of magnetic flux density d ***B*** ^*n*^(***R***_*J*_) ≡ d ***B***_*J*_^*n*^ induced by the current density ***J*** ^***m***_*I*_^ (sustained by nuclear magnetic dipole ***m***_*I*_) within the *n*-electron cloud of a molecule is determined by the differential BSL:^31^

55 $$dB_{J}^{n}=\frac{\mu_{0}}{4\pi }J^{m_{I}}\left(r\right)\times \frac{R_{J}-r}{{\left|R_{J}-r\right|}^{3}}\;d^{3}r=-\kappa^{\mathrm{JI}}\left(r\right)\cdot m_{I}\;d^{3}r$$

where the second identity in this relationship defines a spin–spin coupling density:^32–36^

56 $$\kappa^{J_{\beta }I_{\alpha }}\left(r\right)=-\frac{\mu_{0}}{4\pi }{\text{ϵ}}_{\beta \gamma \delta }\frac{\left(r_{\gamma }-R_{J_{\gamma }}\right)}{{\left|r-R_{J}\right|}^{3}}J_{\delta }^{m_{I_{\alpha }}}\left(r\right)$$

which is expressed in T^2^ J^–1^ m^–3^. Different density functions have been later reported by others.^22–25^ The coupling tensor is evaluated by integrating^57^ eq 56, thus obtaining

$$\kappa^{I_{\alpha }J_{\beta }}=-\frac{\mu_{0}}{4\pi }{\text{ϵ}}_{\alpha \gamma \delta }\int \frac{\left(r_{\gamma }-R_{I_{\gamma }}\right)}{{\left|r-R_{I}\right|}^{3}}J_{\delta }^{m_{J_{\beta }}}\left(r\right)\;d^{3}r=-\frac{\mu_{0}}{4\pi }{\text{ϵ}}_{\beta \gamma \delta }\int \frac{\left(r_{\gamma }-R_{J_{\gamma }}\right)}{{\left|r-R_{J}\right|}^{3}}J_{\delta }^{m_{I_{\alpha }}}\left(r\right)\;d^{3}r$$ 57

The recommended units^45^ of $\kappa^{I_{\alpha }J_{\beta }}$ are 10^19^ T^2^ J^–1^. The interchange theorem embodied in eq 57 is valid for any basis set within a given computational scheme, e.g., coupled Hartree–Fock (CHF), random-phase approximation (RPA) and DFT methods, and it illustrates two possible interpretations of the coupling mechanism. Accordingly, the first (second) line describes it as determined by the current density induced by the magnetic dipole of nucleus *J* (*I*) and acting upon the target magnetic dipole on nucleus *I* (*J*). It is worth recalling that, whereas the tensor in eq 57 may be symmetric in the indices α ↔ β in the presence of molecular symmetries,^5^ the associated density in eq 56 is usually nonsymmetric. Thus, the best representation of the spin–spin coupling is preferably achieved^32,36^ via the superposition of κ^*I*_α_ *J*_β_^(***r***) and κ^*J*_α_ *I*_β_^(***r***), i.e.,

58 $$\kappa_{s}^{I_{\alpha }J_{\beta }}\left(r\right)=\frac{1}{2}\left[\kappa^{I_{\alpha }J_{\beta }}\left(r\right)+\kappa^{J_{\beta }I_{\alpha }}\left(r\right)\right]=-\frac{1}{2}\frac{\mu_{0}}{4\pi }\left[{\text{ϵ}}_{\alpha \gamma \delta }\frac{r_{\gamma }-R_{I_{\gamma }}}{|r-R_{I}{|}^{3}}J_{\delta }^{m_{J_{\beta }}}\left(r\right)+{\text{ϵ}}_{\beta \gamma \delta }\frac{r_{\gamma }-R_{J_{\gamma }}}{|r-R_{J}{|}^{3}}J_{\delta }^{m_{I_{\alpha }}}\left(r\right)\right]$$

The plots of the total SSCC density presented in the Results and Discussion have been obtained by using the isotropic component of the density eq 58. The symmetrized form (eq 58) provides the best description of spin–spin coupling resulting from the interference of two currents, induced by the magnetic dipoles of nuclei *I* and *J*, an expedient adopted also by others.^26^

#### The Spin-Dipolar CDT

According to eqs 33 and 35, the spin-dipolar current density is written^58^

59 $$J_{\alpha }^{m_{I}}\left(r\right)=-\frac{e}{m_{e}}{\text{ϵ}}_{\alpha \beta \gamma }\nabla_{\beta }Q_{\gamma }^{m_{I}}\left(r\right)$$

To obtain the corresponding CDT, $J_{\alpha }^{m_{I_{\delta }}}\left(r\right)$ , let us assume that *Q*_γ_^***m***_*I*_^(***r***) = $Q_{\gamma }^{m_{I_{\delta }}}\left(r\right)$ *m*_*I*_δ__. Using this definition, we obtain

60 $$J_{\alpha }^{m_{I_{\delta }}}\left(r\right)=-\frac{e}{m_{e}}{\text{ϵ}}_{\alpha \beta \gamma }\nabla_{\beta }Q_{\gamma }^{m_{I_{\delta }}}\left(r\right)$$

Thus,

61 $$\left[\begin{array}{l} J_{x}^{\left(2\right)m_{I}}\\ J_{y}^{\left(2\right)m_{I}}\\ J_{z}^{\left(2\right)m_{I}} \end{array}\right]=-\frac{e}{m_{e}}\left[\begin{array}{lll} \nabla_{y}Q_{z}^{m_{I_{x}}}-\nabla_{z}Q_{y}^{m_{I_{x}}} & \nabla_{y}Q_{z}^{m_{I_{y}}}-\nabla_{z}Q_{y}^{m_{I_{y}}} & \nabla_{y}Q_{z}^{m_{I_{z}}}-\nabla_{z}Q_{y}^{m_{I_{z}}}\\ \nabla_{z}Q_{x}^{m_{I_{x}}}-\nabla_{x}Q_{z}^{m_{I_{x}}} & \nabla_{z}Q_{x}^{m_{I_{y}}}-\nabla_{x}Q_{z}^{m_{I_{y}}} & \nabla_{z}Q_{x}^{m_{I_{z}}}-\nabla_{x}Q_{z}^{m_{I_{z}}}\\ \nabla_{x}Q_{y}^{m_{I_{x}}}-\nabla_{y}Q_{x}^{m_{I_{x}}} & \nabla_{x}Q_{y}^{m_{I_{y}}}-\nabla_{y}Q_{x}^{m_{I_{y}}} & \nabla_{x}Q_{y}^{m_{I_{z}}}-\nabla_{y}Q_{x}^{m_{I_{z}}} \end{array}\right]\left[\begin{array}{l} m_{I_{x}}\\ m_{I_{y}}\\ m_{I_{z}} \end{array}\right]$$

#### The Fermi Contact CDT and a Sum Rule

As before, according to eqs 33 and 35, the Fermi contact current density is written^58^

62 $$J_{\alpha }^{m_{I}}\left(r\right)=-\frac{e}{m_{e}}{\text{ϵ}}_{\alpha \beta \gamma }\nabla_{\beta }Q_{\gamma }^{m_{I}}\left(r\right)$$

To obtain the corresponding CDT, $J_{\alpha }^{m_{I_{\delta }}}\left(r\right)$ , let us assume that *Q*_γ_^***m***_*I*_^(***r***) = $Q_{\gamma }^{m_{I_{\delta }}}\left(r\right)$ . From the Fermi Hamiltonian (eqs 18 and 21) and the definition of the Levi–Civita tensor, one can see that only the diagonal elements of matrix $Q_{\gamma }^{m_{I_{\delta }}}$ (i.e., $Q_{x}^{m_{I_{x}}}$ , $Q_{y}^{m_{I_{y}}}$ , and $Q_{z}^{m_{I_{z}}}$ ) are different from 0. Thus, $J_{x}^{m_{I_{x}}}$ = $J_{y}^{m_{I_{y}}}$ = $J_{z}^{m_{I_{z}}}$ = 0, and the Fermi current density becomes

63 $$\left[\begin{array}{l} J_{x}^{\left(3\right)m_{I}}\\ J_{y}^{\left(3\right)m_{I}}\\ J_{z}^{\left(3\right)m_{I}} \end{array}\right]=-\frac{e}{m_{e}}\left[\begin{array}{ccc} 0 & -\nabla_{z}Q_{y}^{m_{I_{y}}} & \nabla_{y}Q_{z}^{m_{I_{z}}}\\ \nabla_{z}Q_{x}^{m_{I_{x}}} & 0 & -\nabla_{x}Q_{z}^{m_{I_{z}}}\\ -\nabla_{y}Q_{x}^{m_{I_{x}}} & \nabla_{x}Q_{y}^{m_{I_{y}}} & 0 \end{array}\right]\left[\begin{array}{l} m_{I_{x}}\\ m_{I_{y}}\\ m_{I_{z}} \end{array}\right]$$

Let us now consider the explicit expression of the perturbed spin-density

64 $$Q_{\alpha }^{m_{J}}\left(r\right)=n\int d\eta_{1}\;dX_{1}\;\left[\Psi_{a}^{\left(3\right)m_{J}*}\left(x_{1},X_{1}\right){ŝ}_{\alpha }\Psi_{a}^{\left(0\right)}\left(x_{1},X_{1}\right)+\Psi_{a}^{\left(0\right)*}\left(x_{1},X_{1}\right){ŝ}_{\alpha }\Psi_{a}^{\left(3\right)m_{J}}\left(x_{1},X_{1}\right)\right]$$

Assuming, for instance, that *Q*_*z*_^***m***_*J*_^ = $Q_{z}^{m_{J_{z}}}$ *m_J_z__* and allowing for the isotropy of space, it follows that $Q_{x}^{m_{J_{x}}}$ ­(***r***) = $Q_{y}^{m_{J_{y}}}$ ­(***r***) = $Q_{z}^{m_{J_{z}}}$ ­(***r***). The interaction energy is

65 $$W^{m_{I}m_{J}}=-\int A^{m_{I}}\cdot J^{m_{J}}\;d^{3}r=-\int A^{m_{I}}\cdot \nabla \times M^{m_{J}}\;d^{3}r=\int_{S}A^{m_{I}}\times M^{m_{J}}\cdot dS-\int M^{m_{J}}\cdot \nabla \times A^{m_{I}}\;d^{3}r$$

The surface integral vanishes because $M^{m_{J}}\left(r\right)\to 0$ for ***S*** → ∞ and

66 $$\nabla \times A^{m_{I}}\left(r\right)=B^{m_{I}}\left(r\right)=\frac{2}{3}\mu_{0}\delta \left(r-R_{I}\right)m_{I}$$

where

67 $$W^{m_{I}m_{J}}=\int M^{m_{J}}\left(r\right)\cdot B^{m_{I}}\left(r\right)\;d^{3}r$$

We have seen that, assuming $Q_{\alpha }^{m_{J_{\beta }}}$ ­(***r***)*m*_*J*_β__, one can only retain diagonal terms $Q_{x}^{m_{J_{x}}}$ ­(***r***)*m*_*J*_*x*__, $Q_{y}^{m_{J_{y}}}$ ­(***r***)*m*_*J*_*y*__, and $Q_{z}^{m_{J_{z}}}$ ­(***r***)*m*_*J*_*z*__. Thus, one obtains the sum rule^35^

68 $$W^{m_{I_{x}}m_{J_{x}}}=\frac{2}{3}\frac{e}{m_{e}}\mu_{0}m_{I_{x}}m_{J_{x}}\int Q_{x}^{m_{J_{x}}}\left(r\right)\delta \left(r-R_{I}\right)\;d^{3}r=\frac{4}{3\hbar }\mu_{0}\mu_{B}m_{I_{x}}m_{J_{x}}Q_{x}^{m_{J_{x}}}\left(R_{I}\right)$$

and analogous results for $W^{m_{I_{y}}m_{J_{y}}}$ and $W^{m_{I_{z}}m_{J_{z}}}$ .

### Implementation at HF and DFT Level of Theory

DFT methods provide a significant improvement of the current density approach to magnetic response properties, with respect to CHF and RPA, as shown in previous papers^59,60^ and reviews.^61–63^ They are also successful for the calculation of NMR coupling constants.^10,64,65^ In this section the operative equations for each contribution will be described.

#### The Diamagnetic Spin–Orbit CDT

The diamagnetic spin–orbit current density tensor defined in eq 46 is the simplest to implement at HF/DFT level of theory. Indeed,

69 $$J_{d_{\alpha }}^{m_{I_{\gamma }}}=\frac{\mu_{0}}{4\pi }\frac{e^{2}}{m_{e}}\frac{\gamma^{\left(0\right)}\left(r\right)}{|r-R_{I}{|}^{3}}\left[\begin{array}{ccc} 0 & -\left(r_{z}-R_{I_{z}}\right) & +\left(r_{y}-R_{I_{y}}\right)\\ +\left(r_{z}-R_{I_{z}}\right) & 0 & -\left(r_{x}-R_{I_{x}}\right)\\ -\left(r_{y}-R_{I_{y}}\right) & +\left(r_{x}-R_{I_{x}}\right) & 0 \end{array}\right]$$

For a closed-shell system, in the one-determinant approximation, assuming real molecular orbitals, we have

70 $$\gamma^{\left(0\right)}\left(r\right)=2\sum_{i=1}^{\mathrm{occ}}\psi_{i}^{\left(0\right)}\left(r\right)\psi_{i}^{\left(0\right)}\left(r\right)$$

The orbitals are expanded as linear combinations of basis functions **χ** according to

71 $$\psi_{i}^{\left(0\right)}\left(r\right)=\sum_{q}C_{\mathrm{qi}}^{\left(0\right)}\chi_{q}\left(r\right)$$

Thus, we obtain

72 $$\gamma^{\left(0\right)}\left(r\right)=2\sum_{\mathrm{pq}}D_{\mathrm{pq}}^{\left(0\right)}\chi_{p}\left(r\right)\chi_{q}\left(r\right)$$

where

73 $$D_{\mathrm{pq}}^{\left(0\right)}=\sum_{i=1}^{\mathrm{occ}}\left[C_{\mathrm{pi}}^{\left(0\right)}C_{\mathrm{qi}}^{\left(0\right)}+C_{\mathrm{pi}}^{\left(0\right)}C_{\mathrm{qi}}^{\left(0\right)}\right]$$

#### The Paramagnetic Spin–Orbit CDT

The paramagnetic spin orbit current density tensor defined in eq 45 can be rewritten as

74 $$J_{p_{\alpha }}^{m_{I_{\beta }}}\left(r\right)=-\frac{\mathrm{ne}}{m_{e}\hbar }R\left\{\int {\left[\sum_{j\neq a}\frac{\left\langle j\left|{\mathrm{B̂}}_{I_{\beta }}^{n}\right|a\right\rangle }{\omega_{\mathrm{ja}}}\Psi_{j}^{\left(0\right)}\left(r,X_{1}\right)\right]}^{*}{\mathrm{p̂}}_{\alpha }\Psi_{a}^{\left(0\right)}\left(r,X_{1}\right)\;dX_{1}+\int \Psi_{a}^{\left(0\right)*}\left(r,X_{1}\right){\mathrm{p̂}}_{\alpha }\left[\sum_{j\neq a}\frac{\left\langle j\left|{\mathrm{B̂}}_{I_{\beta }}^{n}\right|a\right\rangle }{\omega_{\mathrm{ja}}}\Psi_{j}^{\left(0\right)}\left(r,X_{1}\right)\right]\;dX_{1}\right\}$$

where

75 $$\Psi_{a}^{{\left(B_{I}^{n}\right)}_{\beta }}\left(r,X_{1}\right)=\frac{1}{\hbar }\sum_{j\neq a}\frac{1}{\omega_{\mathrm{ja}}}\Psi_{j}^{\left(0\right)}\left(r,X_{1}\right)\left\langle j\left|{\mathrm{B̂}}_{I_{\beta }}^{n}\right|a\right\rangle$$

It is expedient to write eq 75 in the alternative form

76 $$\Psi_{a}^{{\left(B_{I}^{n}\right)}_{\beta }}\left(r,X_{1}\right)=\frac{i}{m_{e}c^{2}}\Psi_{a}^{{\left(E_{I}\times \nabla \right)}_{\beta }}\left(r,X_{1}\right)=\frac{\mu_{0}ie}{4\pi m_{e}}\Psi_{a}^{{\left(\varepsilon_{I}\times \nabla \right)}_{\beta }}\left(r,X_{1}\right)$$

where for the sake of simplicity we have introduced the notation $\varepsilon_{I}=\frac{r_{kI}}{|r_{kI}{|}^{3}}$ . Now, eq 74 becomes

77 $$J_{p_{\alpha }}^{m_{I_{\beta }}}\left(r\right)=\frac{\mu_{0}ne^{2}\hbar }{4\pi m_{e}^{2}}\left\{\int \Psi_{a}^{{\left(\varepsilon_{I}\times \nabla \right)}_{\beta }}\left(r,X_{1}\right)\nabla_{\alpha }\Psi_{a}^{\left(0\right)}\left(r,X_{1}\right)\;dX_{1}-\int \Psi_{a}^{\left(0\right)*}\left(r,X_{1}\right)\nabla_{\alpha }\Psi_{a}^{{\left(\varepsilon_{I}\times \nabla \right)}_{\beta }}\left(r,X_{1}\right)\;dX_{1}\right\}$$

For a closed-shell system, in the one-determinant approximation, assuming real molecular orbitals, we have

78 $$J_{p_{\alpha }}^{m_{I_{\beta }}}\left(r\right)=\frac{\mu_{0}\mu_{B}e}{m_{e}\pi }\sum_{i=1}^{\mathrm{occ}}\left[\psi_{i}^{{\left(\varepsilon_{I}\times \nabla \right)}_{\beta }}\left(r\right)\nabla_{\alpha }\psi_{i}^{\left(0\right)}\left(r\right)-\psi_{i}^{\left(0\right)}\left(r\right)\nabla_{\alpha }\psi_{i}^{{\left(\varepsilon_{I}\times \nabla \right)}_{\beta }}\left(r\right)\right]$$

As usual, the orbitals are expanded as linear combinations of basis functions **χ**, according to

79 $$\psi_{i}^{\left(0\right)}\left(r\right)=\sum_{q}C_{\mathrm{qi}}^{\left(0\right)}\chi_{q}\left(r\right)$$

80 $$\psi_{i}^{{\left(\varepsilon_{I}\times \nabla \right)}_{\beta }}\left(r\right)=\sum_{q}C_{\mathrm{qi}}^{{\left(\varepsilon_{I}\times \nabla \right)}_{\beta }}\chi_{q}\left(r\right)$$

Thus, eq 78 becomes

81 $$J_{p_{\alpha }}^{m_{I_{\beta }}}\left(r\right)=-\frac{\mu_{0}\mu_{B}e}{m_{e}\pi }\sum_{\mathrm{pq}}D_{\mathrm{pq}}^{{\left(\varepsilon_{I}\times \nabla \right)}_{\beta }}\chi_{p}\left(r\right)\nabla_{\alpha }\chi_{q}\left(r\right)$$

where

82 $$D_{\mathrm{pq}}^{{\left(\varepsilon_{I}\times \nabla \right)}_{\beta }}=\sum_{i=1}^{\mathrm{occ}}\left[C_{\mathrm{pi}}^{\left(0\right)}C_{\mathrm{qi}}^{{\left(\varepsilon_{I}\times \nabla \right)}_{\beta }}-C_{\mathrm{pi}}^{{\left(\varepsilon_{I}\times \nabla \right)}_{\beta }}C_{\mathrm{qi}}^{\left(0\right)}\right]$$

is the perturbed density matrix.

#### The Spin-Dipolar CDT

We only need the spin densities induced by the spin-dipolar perturbations (i.e., electric field gradient) and its derivatives. The spin densities can be computed, for a closed-shell system, as

83 $$Q_{\gamma }^{m_{I_{\delta }}}\left(r\right)=\frac{\mu_{0}\mu_{B}}{4\pi }\sum_{i}^{\mathrm{occ}}\left[\psi_{i}^{\left(0\right)}\left(r\right)\psi_{i}^{I_{\gamma \delta }}\left(r\right)+\psi_{i}^{I_{\gamma \delta }}\left(r\right)\psi_{i}^{\left(0\right)}\left(r\right)\right]$$

where ψ_*i*_^*I*_γδ_^ are orbitals perturbed by the electric field gradient integrals centered on perturbing nucleus *I* according to a triplet CKS or CHF scheme. Again, orbitals are expanded as linear combinations of basis functions **χ**, according to

84 $$\psi_{i}^{\left(0\right)}\left(r\right)=\sum_{q}C_{\mathrm{qi}}^{\left(0\right)}\chi_{q}\left(r\right)$$

85 $$\psi_{i}^{I_{\gamma \delta }}\left(r\right)=\sum_{q}C_{\mathrm{qi}}^{I_{\gamma \delta }}\chi_{q}\left(r\right)$$

From the previous expressions we have

86 $$Q_{\gamma }^{m_{I_{\delta }}}=\frac{\mu_{0}\mu_{B}}{4\pi }\sum_{\mathrm{pq}}D_{\mathrm{pq}}^{I_{\gamma \delta }}\chi_{p}\chi_{q}$$

where

87 $$D_{\mathrm{pq}}^{I_{\gamma \delta }}=\sum_{i=1}^{\mathrm{occ}}\left[C_{\mathrm{pi}}^{\left(0\right)}C_{\mathrm{qi}}^{I_{\gamma \delta }}+C_{\mathrm{pi}}^{I_{\gamma \delta }}C_{\mathrm{qi}}^{\left(0\right)}\right]$$

are the perturbed density matrices.

#### The Fermi Contact CDT

The Fermi contact tensor is implemented in a way similar to that used for DSO, PSO, and SD CDTs. We only need the spin density induced by the Fermi contact perturbation and its derivatives. Such a spin density can be computed, for a closed-shell system, as

88 $$Q_{\alpha }^{m_{I_{\alpha }}}\left(r\right)=\frac{2}{3}\mu_{0}\mu_{B}\sum_{i=1}^{\mathrm{occ}}\left[\psi_{i}^{\left(0\right)}\left(r\right)\psi_{i}^{\delta_{I}}\left(r\right)+\psi_{i}^{\delta_{I}}\left(r\right)\psi_{i}^{\left(0\right)}\left(r\right)\right]$$

Again, orbitals are expanded as linear combinations of basis functions **χ**, according to

89 $$\psi_{i}^{\left(0\right)}\left(r\right)=\sum_{q}C_{\mathrm{qi}}^{\left(0\right)}\chi_{q}\left(r\right)$$

90 $$\psi_{i}^{\delta_{I}}\left(r\right)=\sum_{q}C_{\mathrm{qi}}^{\delta_{I}}\chi_{q}\left(r\right)$$

From the previous expressions we have

91 $$Q_{\alpha }^{m_{I_{\alpha }}}\left(r\right)=\frac{2}{3}\mu_{0}\mu_{B}\sum_{\mathrm{pq}}D_{\mathrm{pq}}^{\delta_{I}}\chi_{p}\left(r\right)\chi_{q}\left(r\right)$$

where

92 $$D_{\mathrm{pq}}^{\delta_{I}}=\sum_{i=1}^{\mathrm{occ}}\left[C_{\mathrm{pi}}^{\left(0\right)}C_{\mathrm{qi}}^{\delta_{I}}+C_{\mathrm{pi}}^{\delta_{I}}C_{\mathrm{qi}}^{\left(0\right)}\right]$$

is the perturbed density matrix.

#### Implementation of SSCC Density

Inserting the DSO and PSO current densities (eqs 69 and 81) into the symmetrized BSL (eq 58), the tensors in eqs 23 and 24 are recovered after integration.

If we do the same for the Fermi contact, only the isotropic component is recovered since according to the Poisson equation

93 $$\nabla_{\beta }\frac{r_{\alpha }-R_{I\alpha }}{|r-R_{I}{|}^{3}}=\underset{\mathrm{isotropic}}{\underset{︸}{\frac{4\pi }{3}\delta_{\alpha \beta }\delta \left(r-R_{I}\right)}}-\underset{\mathrm{spurious}}{\underset{︸}{\frac{3\left(r_{\alpha }-R_{I\alpha }\right)\left(r_{\beta }-R_{I\beta }\right)-{\left|r-R_{I}\right|}^{2}\delta_{\alpha \beta }}{{\left|r-R_{I}\right|}^{5}\;}}}$$

all tensor components, resulting after integration of the BSL, contain a spurious term which cancels out calculating the trace. To obtain tensor (eq 26) the spurious term has to be removed as can be worked out by inspecting eqs 8, 9, 12, and 13 of ref 66.

With regard to the spin-dipolar contributions to the coupling density, only the isotropic component is recovered. Analysis on the nonobservable contributions has not been taken into account.

### Standard Response for Triplet States

As regards the calculation of the paramagnetic spin–orbit contribution, the perturbed densities for the three components of the magnetic field at the nuclei has been easily implemented within the standard response program developed so far for the calculation of the electric dipole polarizability density (EDPD) and the specific rotation power density (SRPD).^67^

For the calculation of the perturbed density matrices needed for the determination of FC and SD contributions, which contain the spin operator, we have implemented the resolution of the standard response equation for triplet states. This has been accomplished modifying the construction of the Hessian matrix,^68,69^ which also involves a different way of determining the exchange correlation (XC) term. Here in the following, we report the equations for the calculation of the XC matrix elements directly on the atomic orbital basis, valid up to GGA rung and hybrid GGA.

For a closed-shell system and using the same notation as in ref 67., the calculation of the CKS (or TDKS) exchange-correlation *k*_*pq*_^(1)^ matrix elements is performed using a spin-polarized approach by

94 $$k_{\mathrm{pq}}^{\left(1\right)}=\int \left[\frac{\partial^{2}f^{\mathrm{xc}}}{\partial \gamma_{\alpha }^{\left(0\right)}\partial \gamma_{\alpha }^{\left(0\right)}}-\frac{\partial^{2}f^{\mathrm{xc}}}{\partial \gamma_{\alpha }^{\left(0\right)}\partial \gamma_{\beta }^{\left(0\right)}}\right]\gamma^{\left(1\right)}\chi_{p}\chi_{q}\;d^{3}r+\int \left[\frac{\partial^{2}f^{\mathrm{xc}}}{\partial \gamma_{\alpha }^{\left(0\right)}\partial \gamma^{\left(0_{\alpha },0_{\alpha }\right)}}-\frac{\partial^{2}f^{\mathrm{xc}}}{\partial \gamma_{\alpha }^{\left(0\right)}\partial \gamma^{\left(0_{\beta },0_{\beta }\right)}}\right]\gamma^{\left(0,1\right)}\chi_{p}\chi_{q}\;d^{3}r+\int \left[\frac{\partial^{2}f^{\mathrm{xc}}}{\partial \gamma_{\alpha }^{\left(0\right)}\partial \gamma^{\left(0_{\alpha },0_{\alpha }\right)}}-\frac{\partial^{2}f^{\mathrm{xc}}}{\partial \gamma_{\alpha }^{\left(0\right)}\partial \gamma^{\left(0_{\beta },0_{\beta }\right)}}\right]\gamma^{\left(1\right)}\gamma_{\mathrm{pq}}^{\left(0,0\right)}\;d^{3}r+\int \left[\frac{\partial^{2}f^{\mathrm{xc}}}{\partial \gamma^{\left(0_{\alpha },0_{\alpha }\right)}\partial \gamma^{\left(0_{\alpha },0_{\alpha }\right)}}-\frac{\partial^{2}f^{\mathrm{xc}}}{\partial \gamma^{\left(0_{\alpha },0_{\alpha }\right)}\partial \gamma^{\left(0_{\beta },0_{\beta }\right)}}\right]\gamma^{\left(0,1\right)}\gamma_{\mathrm{pq}}^{\left(0,0\right)}\;d^{3}r+\int \left[2\frac{\partial f^{\mathrm{xc}}}{\partial \gamma^{\left(0_{\alpha },0_{\alpha }\right)}}-\frac{\partial f^{\mathrm{xc}}}{\partial \gamma^{\left(0_{\alpha },0_{\beta }\right)}}\right]\gamma_{\mathrm{pq}}^{\left(1,0\right)}\;d^{3}r$$

where

$$\begin{array}{l} \gamma^{\left(0_{\alpha },0_{\alpha }\right)}=\nabla \gamma_{\alpha }^{\left(0\right)}\cdot \nabla \gamma_{\alpha }^{\left(0\right)}\\ \gamma^{\left(0_{\beta },0_{\beta }\right)}=\nabla \gamma_{\beta }^{\left(0\right)}\cdot \nabla \gamma_{\beta }^{\left(0\right)}\\ \gamma_{\mathrm{pq}}^{\left(0,0\right)}=\nabla \gamma^{\left(0\right)}\cdot \nabla \left[\chi_{p}\chi_{q}\right]\\ \gamma^{\left(0,1\right)}=\nabla \gamma^{\left(0\right)}\cdot \nabla \gamma^{\left(1\right)}\\ \gamma_{\mathrm{pq}}^{\left(1,0\right)}=\nabla \gamma^{\left(1\right)}\cdot \nabla \left[\chi_{p}\chi_{q}\right] \end{array}$$

The first term of eq 94 is the only one needed for LDA functionals; the other ones are needed for GGA functionals. The perturbed probability density and its gradient are

95 $$\gamma^{\left(1\right)}=2\sum_{i}^{\mathrm{occ}}\psi_{i}^{\left(1\right)}\psi_{i}^{\left(0\right)}=2\sum_{i}^{\mathrm{occ}}\sum_{\mathrm{pq}}C_{\mathrm{pi}}^{\left(1\right)}C_{\mathrm{qi}}^{\left(0\right)}\chi_{p}\chi_{q}$$

96 $$\nabla \gamma^{\left(1\right)}=2\sum_{i}^{\mathrm{occ}}\sum_{\mathrm{pq}}C_{\mathrm{pi}}^{\left(1\right)}C_{\mathrm{qi}}^{\left(0\right)}\left[\chi_{q}\nabla \chi_{p}+\chi_{p}\nabla \chi_{q}\right]$$

The LIBXC library^70,71^ has been used for the implementation of the DFT section of the calculation code. The latter is part of the SYSMOIC package.^72^

## Results and Discussion

A few molecules have been considered to provide applications of the theory exposed above for the calculation and visualization of the current density induced by nuclear magnetic dipoles and related indirect spin–spin coupling constant (SSCC) densities. In the following we adopt the acronyms FC, SD, PSO, DSO, and SO for the Fermi contact, spin-dipolar, paramagnetic spin–orbit, diamagnetic spin–orbit, and total spin–orbit contributions, respectively. Owing to the possibility of looking in details at these densities in the real molecular space, we have first considered the ^3^ *J*_HH_ in benzene, ^3^ *J*_PP_ in 1,2-bis­(phosphino)­ethene, and ^3^ *J*_FF_ in 1,2-difluorobenzene. The first of these cases was suggested to be an example of TB interaction, while the second for TS interaction.^21^ The 1,2-difluorobenzene has been taken into account because, as we will show in the following, an interesting comparison with ^3^ *J*_HH_ in benzene can be made. Other molecules from ref 12. were also considered, in particular those for which experimental evidence of interaction TS appears to be the prevailing mechanism.

The calculation protocol we adopted for all molecules can be summarized as (i) geometry optimization at B3LYP/6-31G­(d), only one conformation for each molecule, see below for the specific choices; (ii) solution of the standard response equation^67^ (static case) for gradient vectors related to the nuclear Dirac’s delta function (one component for each nucleus), electric field gradient (six components for each nucleus), and magnetic field (three components for each nucleus) at the B3LYP/6-311+G­(2d,1p) level of approximation. For the traceless electric field gradient, we calculated all the six components instead of five for checking purposes. The diamagnetic spin–orbit contributions were calculated separately for the unperturbed wave functions.

It is emphasized that according to our implementation, the spatial integration of the SSCC densities, related to the FC, SD, PSO, and DSO terms, gives exactly the same numerical results as those obtained after solving the standard response equation taking the average of the tensor diagonal elements, which are obtained contracting the perturbed density matrices with the gradient vectors, as is normally done for the calculation of any second-order response properties. In a number of cases, the superposition of the current densities induced by the coupling nuclear dipoles is shown, which represents the actual physical situation of the static current regime, even if it is not directly related to the corresponding SSCC.

Calculated Ramsey’s contributions to ^3^ *J*_HH_ in benzene, ^3^ *J*_PP_ in 1,2-bis­(phosphino)­ethene, and ^3^ *J*_FF_ in 1,2-difluorobenzene are summarized in Table 1.

**Table 1:** B3LYP/6-311+G­(2d,1p) Spin–Spin Coupling Constants ^3^ *J* in Benzene, Bis­(phosphino)­ethene, and 1,2-Difluorobenzene in Hertz

contrib	^3^ *J*_HH_	^3^ *J*_PP_	^3^ *J*_FF_
FC	6.54	174.66	–2.87
SD	0.05	1.28	11.06
PSO	0.23	–0.07	–27.15
DSO	–0.46	0.014	0.06
total	6.38	175.88	–18.90
exp.	7.56^a^		–21.16^b^

a Average of experimental ^3^ *J*_12_ and ^3^ *J*_34_ of toluene and phenylacetaldehyde from ref 73.

b From ref 74.

For the ^3^ *J*_HH_ of benzene and the ^3^ *J*_PP_ of bis­(phosphino)­ethene, by far the largest contribution to the SSCC is provided by the FC term, i.e., 6.54 Hz with respect to a total of 6.38 Hz for benzene and 175 Hz with respect to a total of 176 Hz for the 1,2-bis­(phosphino)­ethene. For 1,2-difluorobenzene the largest contribution comes from the SO term, the SD is the second in absolute value. The comparison with experimental data available for benzene and difluorobenzene is good enough. In our rather limited experience, we observe that different functionals, i.e., B97-2 and BHandHLYP, provide different results, which sometimes compare better or worse with experimental measurements. As far as we have tested for benzene and difluorobenzene, the B3LYP functional is the best on average.

### ^3^ *J*_HH_ in Benzene

Let us start the analysis of the density functions by first considering benzene.

For ^3^ *J*_HH_ in benzene, the current density induced by one of the magnetic dipoles is shown in the top row of Figure 1, only for the FC contribution on the left and summing all the Ramsey terms on the right. Differences between these two maps are really minimal, in agreement with the dominant role of the FC term also with regards to SSCC density. As can be observed, a typical sequence of current vortices having alternating tropicity is present along the H–C–C–H chain of bonds, very similar to previously reported results for the HH coupling in eclipsed ethane.^32^ Indeed, π-electrons do not contribute on the molecular plane. Starting from a counterclockwise vortex centered on the perturbing dipole, which is set perpendicular to the molecular plane and pointing outward, a clockwise current loop is finally induced on the coupled proton. Since ^3^ *J*_HH_ is positive, this is in agreement with a preferred reversed alignment of the nuclear magnetic dipoles, i.e., the dipole on the coupled proton is pointing inward to have a lower energy, see eq 5.

**Figure 1:**
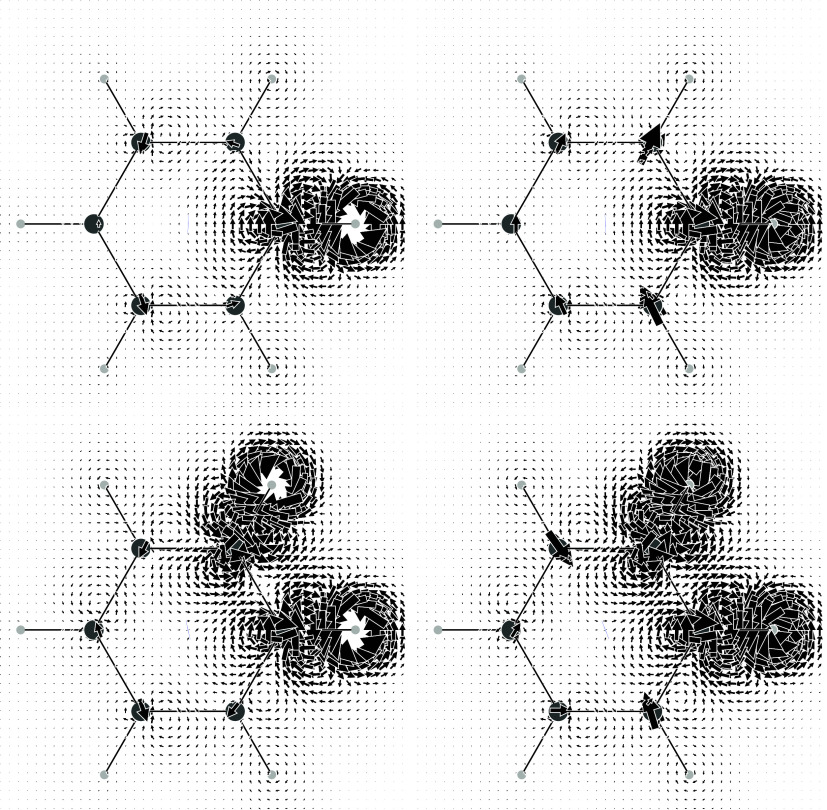
Top row: current density maps induced by a single proton magnetic dipole perpendicular to and facing outward from the molecular plane. Bottom row: superposition of the current densities induced by two antiparallel proton magnetic dipoles at ortho positions. In the left column, only the FC contribution. In the right column, the total FC + SD + PSO + DSO contributions.

The actual physical situation of stationary “dialogue”, in which the coupled nuclei “talk and listen” to one another, is represented in the bottom row of Figure 1, which shows the current density field induced in the electrons by the preferred antiparallel nuclear magnetic dipoles with respect to the parallel disposition. As it is well-known, transitions from both antiparallel and parallel orientations of the magnetic dipoles give rise to the splitting of the NMR signal.

The theory of magnetic, or color, point groups^75–77^ provides useful additional information for interpreting the actual current regime. In particular, the σ_*d*_ plane, which bisects the HCCH bay, cannot be crossed by any current and the two mirror images on its sides present counter tropicity, as clearly illustrated in Figure 1.

In addition to the TB interaction of the proton’s nuclear spins implied in the previous discussion, a TS contribution must also be taken into account. This arises from the clockwise vortex centered on the C–H bond, since, in agreement with the differential BSL (eq 55), this current vortex directly reduces the induced magnetic field at the coupled proton. A similar situation was already accounted for the ^3^ *J*_HH_ in ethane to rationalize the smaller coupling constant calculated for the eclipsed form with respect the staggered one.^32^

However, this is only one side of the story as there are other orientations of nuclear magnetic dipoles that need to be considered. A complete representation can be obtained taking the average of the densities for the diagonal components of the coupling tensor (see eq 58), summing Ramsey’s contribution all together. The total SSCC density for the ^3^ *J*_HH_ coupling in benzene is shown in Figure 2, by means of surfaces for three different values of the scalar field and a diverging color map. Red/blue surfaces enclose regions whose contribution to ^3^ *J*_HH_ is positive/negative. Of course, the spatial integral of the full averaged density equals the value of 6.38 Hz quoted in Table 1

**Figure 2:**
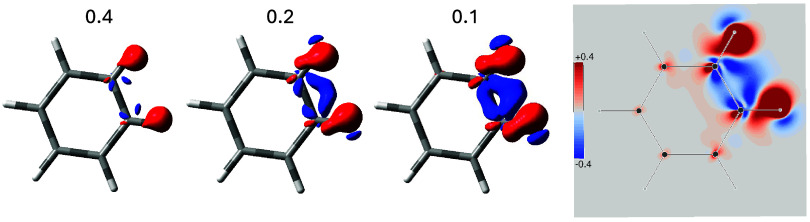
SSCC density for ^3^ *J*_HH_ in benzene, isosurface values are in Hz/*a*_0_^3^.

Although in a different (and more complete) form, the picture that emerges is substantially the same as that we discussed before for the induced current density. The positive contribution has extreme values on the proton and elongates over the C–H bonds. The negative contribution splits into two branches, one parallel to the C–C bond just inside the ring, the other appears outside the ring, but now we notice, especially in the diverging color map, how it propagates to occupy a large part of the bay region. Notably, between the two blue branches there is only a depression that does not contain any red region, confirming the continuity of the two branches. The extension of the blue region into the bay between the two protons makes it even clearer that a TS contribution is also present, although the TB interaction dominates, which is consistent with the positive value of the ^3^ *J*_HH_. For a method to weigh the TS and TB contributions (see ref 20).

### ^3^ *J*_FF_ in 1,2-Difluorobenzene

The substitution of two hydrogens with a pair of fluorine atoms provides a completely different picture for the ^3^ *J*_FF_. As shown in Table 1, the calculated FC (−3 Hz) is in absolute value the smallest compared to SD and SO (11 and −27 Hz, respectively).

The current density induced by one ^19^F magnetic dipole is shown on the left of Figure 3. The current regime about the perturbing dipole features a pair of nested circulations, with an internal counterclockwise circulation surrounded by a clockwise circulation of lesser intensity. It can be noted that the induced current around the second fluorine nucleus at ortho position has the same tropicity as the innermost more intense current of the perturbing dipole. Therefore, the two nuclear magnetic dipoles prefer a parallel disposition to have a lower energy according to eq 5. The stationary total current density for such physical orientation of the dipoles is shown on the right of Figure 3.

**Figure 3:**
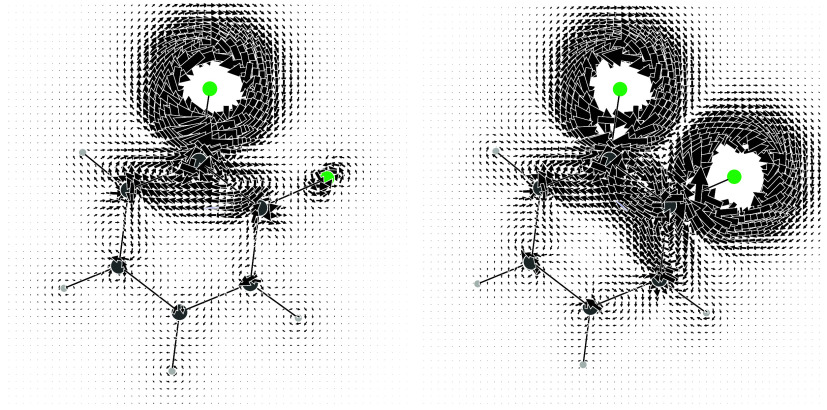
On the left: total current density map induced by a single ^19^F magnetic dipole perpendicular to and facing outward from the molecular plane. On the right: superposition of total current densities induced by two parallel ^19^F magnetic dipoles at ortho positions. Total current densities have been obtained by summing FC + SD + PSO + DSO contributions.

Also in this case, the theory of magnetic point groups^75–77^ provides some useful information. In particular, the σ_*d*_ plane, bisecting the FCCF bay, is now associated with the time reversal operator, i.e., *R*σ_*d*_,^32^ which implies that it can be crossed only perpendicularly by the current. When the current approaches the symmetry plane with an angle not equal to π/2, the phase portrait of a saddle lying on *R*σ_*d*_ along with two vortex centers can be observed in Figure 3.

The actual shape of the various SSCC densities is reported in Figure 4. As can be observed, there are regions where the FC contribution is rather large, at least as much as the SO in absolute value. In contrast, the SD contribution is markedly smaller along the bonds and in the bay region between the two fluorine atoms. Looking at the disposition of both FC and SO densities around the bonds with that found in the bay region between the fluorine atoms, one can easily appreciate that in this case the interaction occurs mainly TS. Very interesting is the correspondence between the region containing the saddle and the vortex centers, shown in Figure 3, with the surface perforation that occurs near the middle of the C–C bond outside the benzene ring, see bottom row of Figure 4.

**Figure 4:**
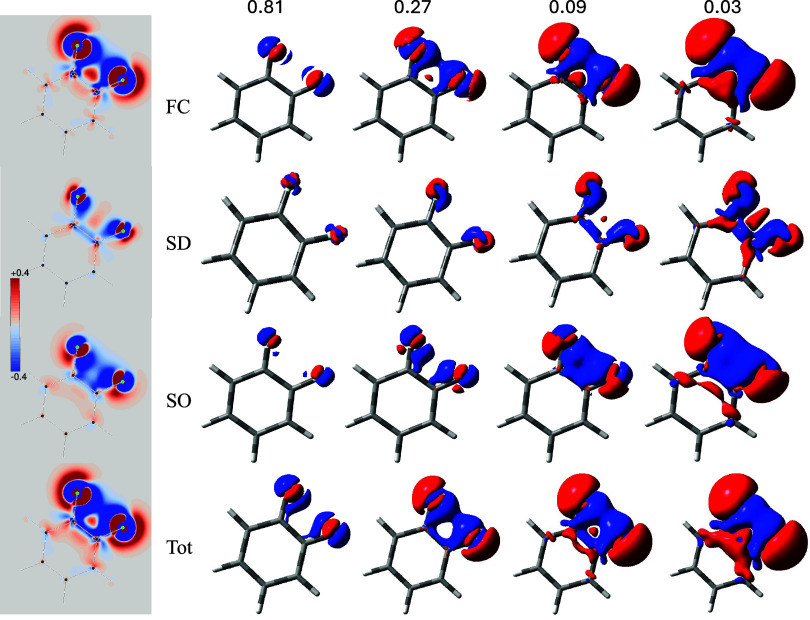
SSCC densities for ^3^ *J*_FF_ in 1–2-difluorobenzene, isosurface values are in Hz/*a*_0_^3^.

Unlike the ^3^ *J*_HH_ density in benzene, the ^3^ *J*_FF_ density shows a change of sign that marks a sharp break between the bay and the C–C bond region.

The TS interaction for ^3^ *J*_FF_ in 1,2-difluorobenzene is clearly promoted by the proximity of the two nuclei and the electron density available between them. In fact, if we consider the SSCC densities calculated for the ^4^ *J*_FF_ in 1,3-difluorobenzene, shown in Figure 5, and ^5^ *J*_FF_ in 1,4-difluorobenzene, shown in Figure 6, a substantially complete TB interaction can be observed. In both cases, the SSCC density lies along the molecular structure of the bonds showing a persuasive characteristic alternation of sign.

**Figure 5:**
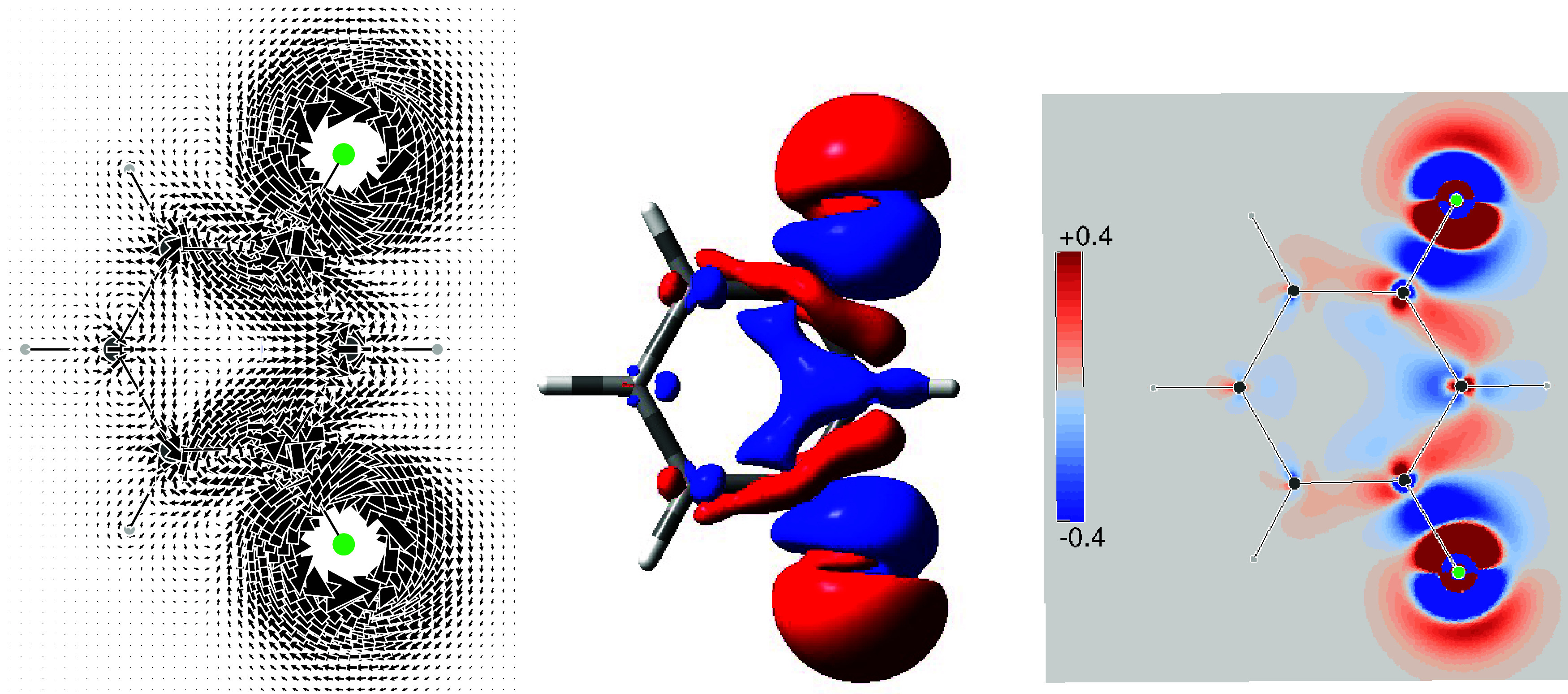
1,3-Difluorobenzene. (left) Superposition of all-Ramsey contributions to the current densities induced by two antiparallel ^19^F magnetic dipoles. (center, right) Total SSCC density for ^4^ *J*_FF_. The isosurface value is 0.03 Hz/*a*_0_^3^.

**Figure 6:**
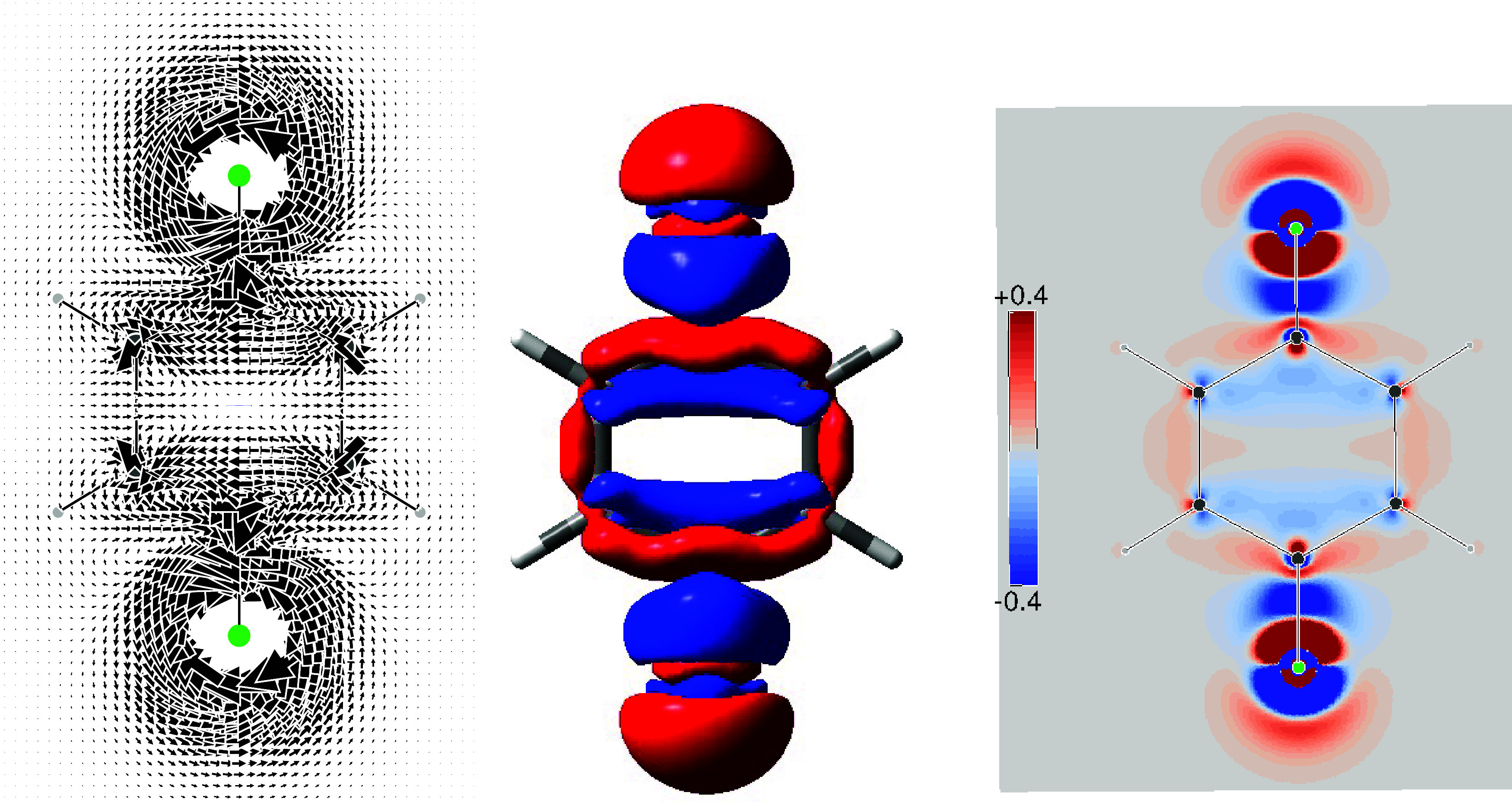
1,4-Difluorobenzene. (left) Superposition of all-Ramsey contributions to the current densities induced by two antiparallel ^19^F magnetic dipoles. (center, right) Total SSCC density for ^5^ *J*_FF_. The isosurface value is 0.03 Hz/*a*_0_^3^.

Spatial integration of the SSCC densities shown in Figures 5 and 6 gives total coupling constants in good agreement with experimental values found in the literature, see Table 2, confirming the good quality of the calculations. However, the dissection into Ramsey’s contributions shows a rather complex situation, i.e., although both ^4^ *J*_FF_ and ^5^ *J*_FF_ are attributable to a TB interaction, they do not share the prevailing contributions; indeed, the SO contribution dominates the F–F coupling in *m*-difluorobenzene, while SD and FC dominate for the *para* isomer.

**Table 2:** B3LYP/6-311+G­(2d,1p) Spin–Spin Coupling Constants in *m*- and *p*-Difluorobenzene in Hertz

contrib	^4^ *J*_FF_	^5^ *J*_FF_
FC	–0.76	7.26
SD	–0.17	15.35
PSO	5.94	–1.61
DSO	–1.01	–1.05
total	4.00	19.95
exp.	5.8^a^	18.1,^a^ 17.1^b^

a Unperturbed coupling *J*_0_ from ref 78.

b From ref 79.

### ^3^ *J*_PP_ in Bis­(phosphino)­ethene

As previously mentioned, the ^3^ *J*_PP_ in bis­(phosphino)­ethene was suggested to be an example of TS interaction.^21^ It should be noted that the conformation chosen for the molecule, with the two phosphino groups bisected by the symmetry plane of ethylene and with hydrogen atoms directed outward, is not the equilibrium one. In this way, the lone pairs of the phosphorus atoms are facing each other, a condition that should favor TS interaction. Adopting a different method from ours and considering only the FC contribution, the TS transmission pathway was estimated to contribute for more than 90% of the final SSCC.^20^

Our result is given in Figure 7, where the total SSCC density is displayed for a set of decreasing isosurface values. A clean TS transmission path can be observed, accompanied by a TB path which starts to be visible for the lowest density value. As for 1,2-difluorobenzene, the surface is perforated near the center of the C–C bond, in correspondence with a zone of saddle points of the stationary total current density for the preferred physical situation represented by two antiparallel ^31^P magnetic dipoles, in agreement with the positive value of the coupling constant and eq 5 (see Figure 8). This interruption separates the two transmission paths and seems to represent a repetitive motif that characterizes situations in which the two types of interaction are simultaneously present, even if to a different extent.

**Figure 7:**
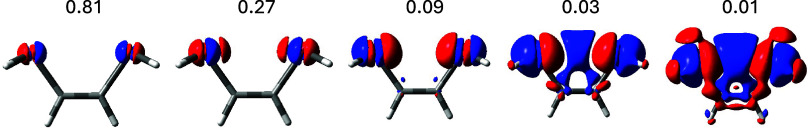
Total SSCC density for ^3^ *J*_PP_ in bis­(phosphino)­ethene, isosurface values are in Hz/*a*_0_^3^.

**Figure 8:**
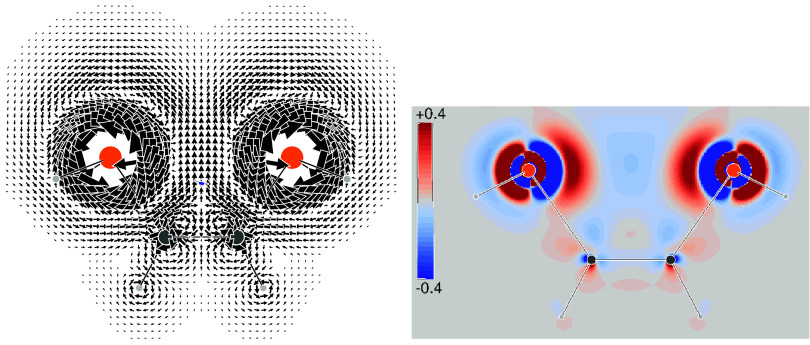
Bis­(phosphino)­ethene. (left) Superposition of all-Ramsey contributions to the current densities induced by two antiparallel ^31^P magnetic dipoles. (right) Total SSCC density for ^3^ *J*_PP_.

### Three More Cases of Through-Space Interaction

By examining the suggestive papers of Buckingham and Cordle^12^ one can glean many hints about TS nuclear spin–spin interaction. In particular, keeping the enumeration given in ref 12, we focused our attention on 4,5-difluoro-1-methylphenanthrene (**IX**), 2,2′-difluorobiphenyl (**X**), and *trans*-1.1′-difluorotetrabenzopentafulvalene (**XIX**), which we believe to be good cases of study to test our procedure.

Calculated SSCC values are reported in Table 3. As can be seen, the dissection in Ramsey’s contributions reveals that the FC is the largest in all cases, even if the SO term in **IX** and **X** is not at all negligible. In these cases, at least, SD does not seem to provide a significant contribution. The comparison with the available experimental results is satisfactory for **IX** and **XIX**, while for **X**, which can easily rotate about the central bond, the discrepancy can be attributed to a smaller F···F distance determined by the geometry optimization we did for the free molecule.

**Table 3:** B3LYP/6-311+G­(2d,1p) ^5^ *J* and ^7^ *J* Spin–Spin Coupling Constants in 4,5-Difluoro-1-methylphenanthrene (**IX**), 2,2′-Difluorobiphenyl (**X**), and *trans*-1,1′-Difluorotetrabenzopentafulvalene (**XIX**) in Hertz

contrib	^5^ *J*_FF_ in **IX**	^5^ *J*_FF_ in **X**	^7^ *J*_HF_ in **XIX**
FC	197​	49.​2	10.​1
SD	1.​4	1.​6	–0.​4
SO	–29​	–9.​6	0.​2
total	169​	41.​1	9.​9
exp.	170​^a^	16.​5^a^	7​^b^

a From ref 80.

b From ref 81.

Total SSCC densities are shown in Figures 9–11 for **IX**, **X**, and **XIX**, respectively. In each figure, the SSCC is represented by a set of surfaces for six decreasing density values, so that one can more easily appreciate the places where the density is most conspicuously present. For all three molecules the SSCC density is mainly located between the coupling nuclei, clearly evidencing a TS interaction. Although the theoretical ^5^ *J*_FF_ in **IX** is four times (10 times the experimental) larger than in **X**, it is difficult to find such a large difference by comparing the densities between the two series of surfaces. This is not surprising since the major contribution comes from the FC term, which is mainly observed on the nuclei. A TB contribution of some magnitude is found for both ^5^ *J*_FF_. Looking carefully, a faint TB contribution can be seen also for the ^7^ *J*_FH_, which is one of the largest seven-bonds H···F coupling known.

**Figure 9:**
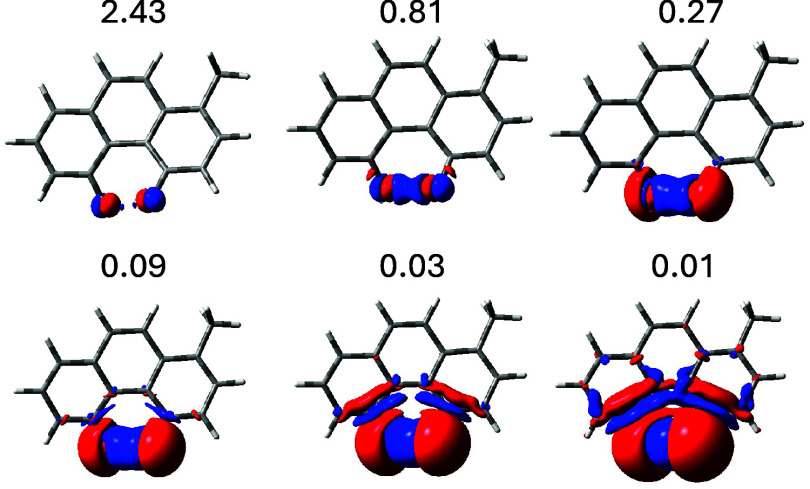
Total SSCC density for the ^5^ *J*_FF_ in 4,5-difluoro-1-methylphenanthrene. Isosurface values are in Hz/*a*_0_^3^. Red indicates positive and blue negative. The internuclear distance is 2.406 Å.

**10:**
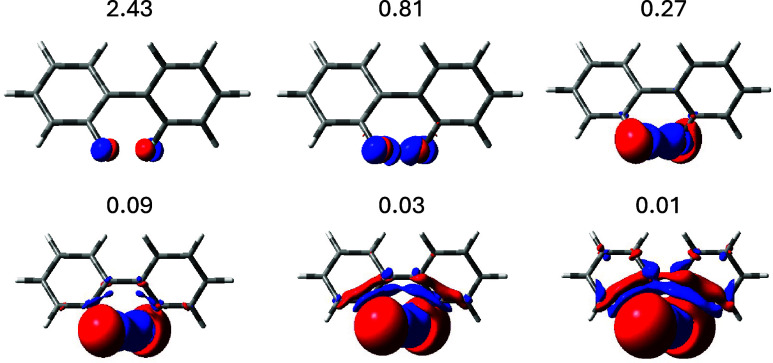
Total SSCC density for the ^5^ *J*_FF_ in 2,2′-difluorobiphenyl. Isosurface values are in Hz/*a*_0_^3^. Red indicates positive and blue negative. The internuclear distance is 2.673 Å.

**Figure 11:**
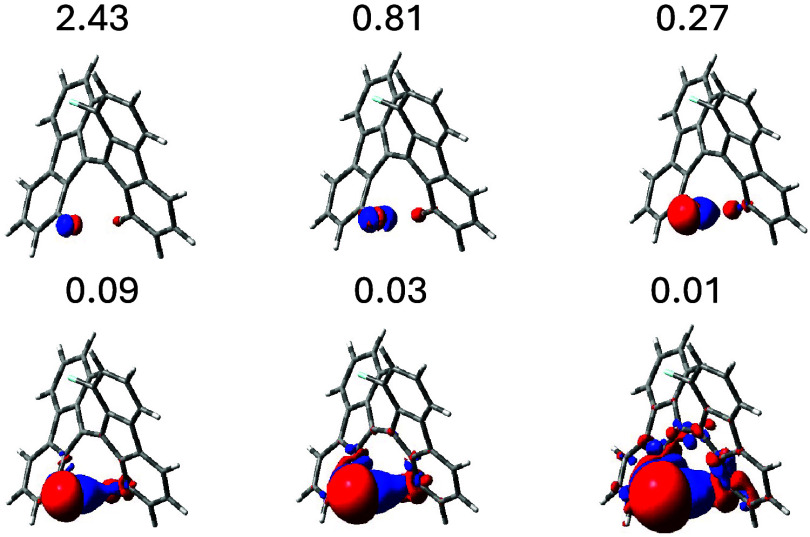
Total SSCC density for the ^7^ *J*_FH_ in *trans*-1.1′-difluorotetrabenzopentafulvalene. Isosurface values are in Hz/*a*_0_^3^. Red indicates positive and blue negative. The internuclear distance is 2.476 Å.

### Remarks on Sign and Sign Patterns

The absolute sign of the coupling constant can be obtained by the methods described by Buckingham and Lovering.^82^ Despite the limited number of cases examined here, some striking features seem to be present. First, in the central region of the TS interaction, the SSCC density is always negative, regardless of the type of the coupling atoms. This feature is confirmed also at the relativistic DFT level.^26^ For ^3^ *J*_FF_ in 1,2-difluorobenzene, this negative sign is determined by both the FC and SO contributions, see Figure 4. This does not imply that the sign of the total spin–spin coupling constant, including all Ramsey terms, is negative; the 4,5-difluoro-1-methylphenanthrene provides a typical example. However, we notice a recurring pattern, i.e., an extended portion in the center of the TS path is characterized by a negative SSCC density, which might influence the total sign of the coupling constant.

Second, the SSCC density often changes sign along both the TS and TB paths. While this can be traced back to the HDVV model, it should be noted that the sign oscillates heavily especially close the coupling nuclei, where the number of times it changes can be associated to the row of the element, as can seen looking at the SSCC density close to H (one time), F (two times), and P (three times). However, as reported in ref 83: “...it must be emphasized that the Dirac vector model mentioned here is a gross oversimplification of the real situation, and many conclusions based on it are found to be incorrect”. The present paper shows the limits of any naive approach relying on the HDVV model.

## Conclusions

The indirect spin–spin coupling density function, expressed in terms of the current density induced by the nuclear magnetic dipoles, has been reformulated in a new comprehensive way and implemented at HF and DFT level (GGA and hybrid GGA), within the SYSMOIC package as a new option of the standard linear response code.

It is shown that the SSCC density function is a valuable tool to analyze the regions of the molecular space involved in the interaction path between the coupling nuclei. Although only a few molecules have been studied, the results arrived at show the effectiveness of the method, as they allow us to concretely visualize what is meant by TB and TS interaction.

Quite often the FC contribution is the leading term, but this is not generally true, see, for example, the 1,3-difluorobenzene. Therefore, including all four terms in the spin–spin coupling density function represents a step forward in understanding the spin polarization pattern.
